# Applications of Plasma-Liquid Systems: A Review

**DOI:** 10.3390/ma12172751

**Published:** 2019-08-27

**Authors:** Fatemeh Rezaei, Patrick Vanraes, Anton Nikiforov, Rino Morent, Nathalie De Geyter

**Affiliations:** 1 Research Unit Plasma Technology (RUPT), Department of Applied Physics, Faculty of Engineering and Architecture, Ghent University, St-Pietersnieuwstraat 41 B4, 9000 Ghent, Belgium; 2 Research group PLASMANT, Department of Chemistry, University of Antwerp, Universiteitsplein 1, 2610 Wilrijk, Belgium

**Keywords:** plasma-liquid interactions, nanomaterial processing, analytical chemistry, water treatment, sterilization and biomedicine, cancer therapy, agriculture and food safety, oil treatment, polymeric solution treatment

## Abstract

Plasma-liquid systems have attracted increasing attention in recent years, owing to their high potential in material processing and nanoscience, environmental remediation, sterilization, biomedicine, and food applications. Due to the multidisciplinary character of this scientific field and due to its broad range of established and promising applications, an updated overview is required, addressing the various applications of plasma-liquid systems till now. In the present review, after a brief historical introduction on this important research field, the authors aimed to bring together a wide range of applications of plasma-liquid systems, including nanomaterial processing, water analytical chemistry, water purification, plasma sterilization, plasma medicine, food preservation and agricultural processing, power transformers for high voltage switching, and polymer solution treatment. Although the general understanding of plasma-liquid interactions and their applications has grown significantly in recent decades, it is aimed here to give an updated overview on the possible applications of plasma-liquid systems. This review can be used as a guide for researchers from different fields to gain insight in the history and state-of-the-art of plasma-liquid interactions and to obtain an overview on the acquired knowledge in this field up to now.

## 1. Introduction

**Figure 1:**
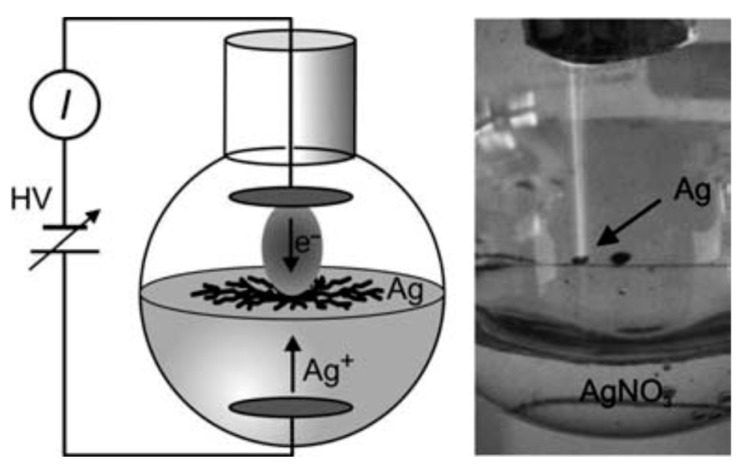
Set-up of the reproduced Gubkin’s experiment: Silver is dissolved at the anode placed in the liquid electrolyte and reduced at the plasma-electrolyte interface. Reprinted with permission from [3].

From a historical point of view, it can be said that the first report on the possibility of the interaction of plasma with liquids dates back to 1789 when van Troostwijk and Deinman [1] reported the decomposition of water by an electric discharge. However, it was Gubkin [2] in 1887 who pioneered electrical discharge interactions with liquids using Glow Discharge Electrolysis (GDE) as a unique electrochemical technique. Gubkin used a glow discharge to reduce metallic salt (silver ions Ag^+^) in an aqueous solution of AgNO_3_. In this technique, the sample solution usually acts as the cathode and the discharge is generated between the metal anode and the liquid cathode by applying high voltage. Gubkin observed the deposition of visible metal particles formed through the reduction of the metal cations by interaction with free electrons from the plasma discharge at the discharge-liquid interface. Figure 1 shows Gubkin’s experimental set-up, as reproduced by Janek et al. [3]. Gubkin’s inspiring work was a startup for a new research field which was later called plasma electrochemistry. In fact, this inspiring work was performed long before Irving Langmuir proposed the term “plasma” in his paper in 1928 [4] to describe the positive column of a low pressure gas discharge. However, the luminous discharge between two carbon electrodes was already reported by two French physicists, Hippolyte Fizeau and Leon Foucault in 1844 [5].

After Gubkin, much attention was given to this new research field for the development of the GDE technique and for investigation of micro, spark, or arc discharges in liquid electrolytes [6,7,8,9]. Some groups, mainly Klemenc et al. and Brenner et al. [10,11,12,13], reproduced and improved Gubkin’s simple experiment in the 1950s and 1960s. Also, some studies followed on material synthesis from plasma-liquid interactions (PLIs) [14,15,16,17,18,19]. To this date, this method is still widely used for the synthesis of nanoparticles (NPs) and nano-structured materials at the plasma-liquid interface [20]. One of the interesting experiments related to PLIs is Miller’s experiment on origins of life in 1953 [21]. He used water, methane, ammonia, and hydrogen which were sealed inside a glass flask. The heated water evaporated and entered a larger flask where continuous electrical sparks were fired between the electrodes to simulated lightening in the water vapor and gaseous mixture.

To understand the fundamental physical aspects of PLIs, many groups have studied the formation and propagation of electrical discharges in different organic solvents and dielectric liquids. Although the study on the mechanisms of electrical breakdown of dielectric liquids (mostly insulating oils such as those used for transformers, capacitors, etc.) had started from the 1940s [22,23,24], these mechanisms have been more extensively investigated from the 1970s [25,26,27,28,29,30]. However, the chemical complexity and variety of insulating oils increases the difficulty in understanding the basic breakdown mechanisms. Therefore, many studies have been conducted using pure insulating liquids with a simple chemical structure such as organic solvents and liquefied gases [31,32]. In one of the early studies, Komelkov et al. [22] investigated the breakdown of transformer oil and water and they found that spark propagation velocity in water is higher than that in transformer oil (ε = 2). Moreover, it was reported that the spark in water emits much more intense light. Subsequently, many investigations have been done to understand pre-breakdown and breakdown phenomena in water (with a large dielectric constant, ε = 80) [23,24,33,34,35,36,37,38,39,40]. Due to its polarity and conductivity, pre-breakdown and breakdown phenomena in water differs from those in organic solvents or insulating oils. However, it has been shown that in both cases, the streamers form as precursor to breakdown [41]. In 1932, four years after Irving Langmuir coined the term “plasma”, Carter and Campbell [42] published a report on investigations of arc discharges in water including descriptions of the chemical nature of the arc-produced byproducts. Another considerable study of underwater arcs (usually produced by capacitive discharges in water) was the 1960 publication by Martin [43] which considered the plasma properties of such discharges.

The capability of plasma technology in plasma-liquid systems which had been confirmed due to the results of previous studies, along with the crucial issue of water pollution as well as the lack of potable water, have led to the emergence of extensive studies focusing on the use of various types of discharge plasmas for water purification and wastewater treatment applications from 1973 onward [44,45,46,47,48,49]. Additionally, many research groups also dealt with the fundamental physical and chemical properties of plasma-water systems. In the 1980s, Clements et al. [41] investigated the pre-breakdown phenomena in water when using point-plane streamer-corona discharge geometry. In the following years, Sun et al. [50], Sunka et al. [51], and Joshi and Locke et al. [52] investigated the generation of chemically active species by pulsed streamer-corona discharges in water. Besides water purification and wastewater treatment, different studies have also been carried out using PLIs for other interesting applications mainly for medical applications, NPs and nanomaterials synthesis, the food industry, and, very recently, treatment of pre-electrospinning polymer solutions.

Although there are some excellent review papers in this field, in particular reviews by Malik et al. [46], Bruggeman et al. [53,54], Lesaint [55], Qiang et al. [56], and Locke et al. [57,58], an updated review considering the possible applications is of importance. Due to the multidisciplinary character of this research topic, as well as its broad range of applications, such a review will be useful as a guide for both plasma scientists and researchers from many different non-plasma fields. The present review aims to address this need, by giving an overview on the various applications of plasma-liquid systems, including the optimization of transformer oil, polymer solution treatment, nanoparticle synthesis, analytical chemistry, organic and inorganic water pollutant removal, sterilization, plasma medicine, agricultural applications, and food treatment.

## 2. Plasma

**Figure 2:**
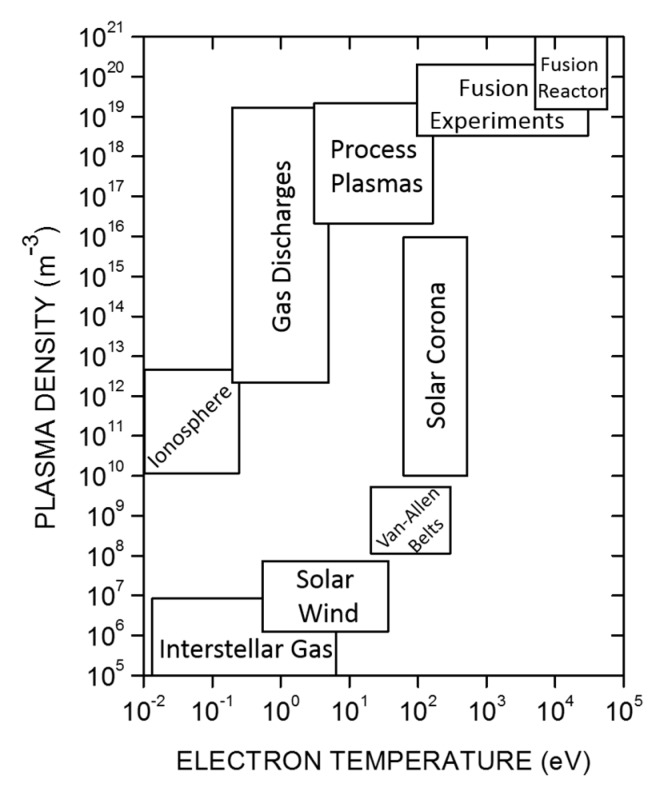
Plasma classification based on electron temperature and plasma density, adapted from [60].

Simply speaking, plasmas are quasi-neutral ionized gases. Hence, they consist of positive and negative ions, electrons, free radicals, photons, metastables as well as excited and neutral atoms and molecules. Plasmas can be classified by gas temperature, electron and ion temperatures and densities, pressure, current magnitude, powering mode, thermodynamic equilibrium and ionization degree (i.e., the ratio of the number density of charged particles to the total number density of species including neutrals and charged particles). An example of such a classification is given in Figure 2. The ionization degree of plasma can vary from partially ionized (e.g., 10^−4^ to 10^−6^) to completely ionized. Completely ionized plasmas can be found in much of the visible matter in the universe such as stars and visible interstellar matter. They are encountered in many forms, from the low pressure plasma in the interstellar medium to the high pressure and highly energetic fusion processes in the core of the sun. While such astrophysical plasmas have been extensively studied and described in the past and the 21st century, a lot of interest has been directed to gas discharges at laboratory scale as well. Besides the astrophysical plasmas, one can also find completely ionized plasmas in laboratories involved in nuclear fusion research and for thermonuclear systems including tokamaks, stellarators, plasma pinches, and so on [59].

A classification can be also made for plasmas based on the conditions of thermodynamic equilibrium: Equilibrium and non-equilibrium plasmas, also often termed thermal and non-thermal plasmas, respectively. This classification is related to the energetic levels of electrons and the heavy species of the plasma. Equilibrium plasmas which are often obtained at high pressure (≥10^5^ Pa) approach a local thermodynamic equilibrium (LTE) between electrons and the other species. This implies that all plasma components (electrons, ions, neutral species, etc.) have nearly the same temperature (T_e_ ≈ T_ion_ ≈ T_gas_), typically ranging from 4 × 10^3^ to 20 × 10^3^ K which is much higher than the ambient temperature. On the other hand, non-equilibrium plasmas are usually obtained at atmospheric pressure or lower. For these plasmas, electron temperature is in the range of 10^4^ to 10^5^ K, whereas the other plasma species are at temperatures close to the ambient temperature. This is due to the long mean free paths between electrons and heavy plasma species. Therefore, due to the inefficient energy transfer, thermal equilibrium cannot be achieved, resulting in an electron temperature much higher than the temperature of the other species in the plasma (T_e_ >> T_ion_ ≈ T_gas_ ~ 300–400 K).

In the category of non-thermal (or cold) plasmas, atmospheric pressure plasmas have gained considerable interest. This type of plasma is near standard conditions for temperature and pressure (P ≈ 1 atm, T_gas_ ≈ 300 K). While its gas temperature is kept relatively low, gas molecules in this plasma are excited or ionized by accelerating electrons to sufficiently high velocities. Since its electron temperature is always much higher than its gas temperature, it is usually included in the category of the non-thermal plasmas. These non-thermal atmospheric pressure plasmas are of particular technical and industrial interest as they can operate in ambient air, avoid undesired gas heating and do not require extreme handling conditions [61,62]. Moreover, with an appropriate electron energy distribution, desired plasma reactions can be specifically triggered. That is, energy does not need to be spread without profit into all degrees of freedom such as into the thermal motion, rotation, and vibration of neutral gas molecules but only into those degrees of freedom that efficiently generate the desired reaction products for the intended application. In this manner, generated plasmas can be turned into a specialized tool. Energy can be channeled into desired excitations and reactions by variation of gas composition, electrode shape, dimensions, circuit characteristics, and other operational parameters. For these reasons, non-thermal atmospheric pressure plasmas in and in contact with liquids are the main focus of the present review.

## 3. Applications of Plasma-Liquid Systems

### 3.1. Plasma in Nanomaterial Processing

Today, various methods are available for NP synthesis, among which reduction of metal ions in solutions is a very powerful tool for fabricating NPs with different sizes, shapes, and compositions [56]. Wet-chemical methods, which are based on chemical reduction occurring by mixing a suitable reducing agent into the solution, is still an effective route. However, the wet-chemical reduction method suffers from long processing times, usually up to several hours. To overcome this problem, many groups have attempted to develop alternative reduction methods in which energetic electrons serve as reducing agent. Due to the unique properties of plasma and also because of the metal ions dissolubility in the liquids, plasmas in liquids or in contact with liquids have attracted significant attention for NP synthesis. NP synthesis using plasma-liquid interfaces presents some important advantages such as the unnecessity of using reducing agents, the simplicity of its experimental design, and also the continuous synthesis during plasma irradiation [63,64,65,66,67]. Reducing agents can be directly produced during the PLIs-based NP synthesis process, which is a key advantage of this technology in contrast to the conventional wet-chemical solution-based methods.

In nanomaterial processing using PLIs, the most attention is paid to reactions occurring at the plasma-liquid nano-interface [68]. Indeed, NP formation is most probably attributed to the complicated physical and chemical reactions occurring in the plasma-liquid interface such as reduction, oxidation, and sputtering. There have been several computational investigations that have discussed the processes occurring at the plasma-liquid interface and how their fluctuations affect the results of material processing as well. For example, it has been shown by Shirafuji et al. [68,69,70] using numerical simulation that slow liquid ions tend to remain on the top of the liquid surface in contact with plasma when the plasma is generated by an AC-driven dielectric barrier discharge (DBD). This tendency implies that slow liquid ions preferentially interact with the species supplied from the gas phase plasma and govern reactions to generate final products in the liquid phase.

Although the capability of plasma-based nanomaterial processing has been confirmed by numerous studies [71,72,73,74,75], the synthesis of NPs with a defined shape is still very challenging and often not very reproducible. Therefore, a precise control over the synthesis rate and the NP morphology remains unattainable because the inevitable high voltage discharges at atmospheric pressure and the dynamic behavior of the plasma-liquid interface prevent the analysis of the precise plasma properties in the interfacial region [76]. To deal with this problem, ionic liquids have been proposed as the most suitable liquids for the plasma-assisted formation of NPs [77,78,79,80,81,82,83,84,85,86,87] due to their composition (consisting of only positive and negative ions), low vapor pressure, high heat capacity and non-flammability. These characteristics, in contrast to volatile liquids or water, enable the introduction of ionic liquids to vacuum plasma systems and therefore do not limit the use to only plasmas operated at high pressures. Moreover, in case of low pressure plasmas, nanomaterial synthesis is not limited to a small sized plasma region, which typically enhances the yield of produced nanomaterials. Using ionic liquids, Brenner et al. [13] could obtain dendrites of several metals in a vacuum glow discharge system. Additionally, in 1998, Kawamura et al. [88] synthesized micro-scaled fine Ag particles in an atmospheric pressure DC plasma system. These authors used a molten LiCl-KCl-AgCl salt as anode, because the electrons generated from the plasma cathode can reduce any metal ion in its molten form. Based on this result, the same approach, using plasma as cathode and the ionic liquid as anode, has been applied in many cases for NP synthesis [76,78,87,89,90].

**Figure 3:**
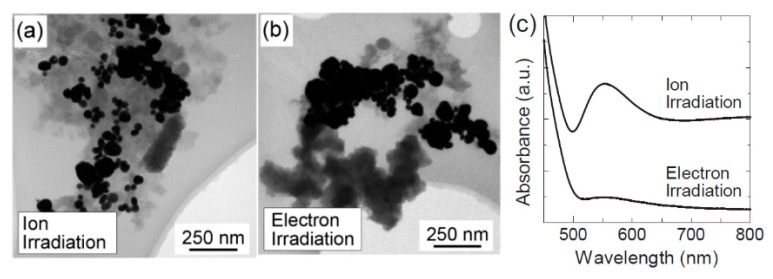
Transmission electron microscopy (TEM) images of gold NPs synthesized in (**a**) the ion irradiation mode and (**b**) the electron irradiation mode; (**c**) UV-vis absorption spectra of gold NPs. Reprinted from [76].

Although ionic liquids present some advantages, using them makes it difficult to eliminate the conjugation of the ionic liquids from the surface of NPs when it is desired to change the surface function of NPs. Also, the solubility of many metal salts is low in ionic liquids in comparison to water. Moreover, the reducing species produced by plasma are usually more efficient in water-based solutions than in the ionic liquid-based ones, due to their better mobility in water-based solutions [56]. Consequently, numerous studies have also focused on plasma-assisted NP synthesis using water-based solutions, of which a few examples are given in Table 1. Using PLIs technology, significant attention has been paid to the fabrication of gold NPs. For this purpose, a multitude of plasma systems were used including DC discharges [76], atmospheric micro-plasmas [75,91], pulsed DC discharges [64,92,93,94] and DC arc discharges [95]. To fabricate gold NPs, Kaneko et al. [76] generated a DC discharge on an ionic liquid and studied both ion and electron irradiation modes combined with the introduction of ionic liquids under strong magnetic fields up to several tesla. The authors concluded that ion irradiation is more effective for the synthesis of gold NPs compared to the conventional electron irradiation system (see Figure 3). They also observed that corresponding to the shape of the strongly-magnetized plasma, periodic or ring shaped gold NP structures could be formed. Mariotti et al. [91] also examined the synthesis of gold NPs, but used a DC micro-plasma. These authors especially considered electron-induced reactions to determine the chemical reactions occurring in the liquid phase. Their results confirmed that the synthesis of gold NPs is achieved without the use of any surfactant or any reagent other than the gold precursor.

Besides gold NPs, silver NPs have also been widely synthesized using different plasma discharge regimes, as can be seen in Table 1. Yu-Tao et al. [72] reported the synthesis of Ag NPs with a mean diameter of 3.5 nm using an Argon (Ar) DBD plasma jet applied on a solution containing AgNO_3_. These researchers used ethanol as a solvent and reducing agent because, in case of using water, the discharge would transit to an arc mode and the temperature would increase too much. Additionally, polyvinyl pyrrolidone (PVP) was used as macromolecular surfactant and a pulsed power supply was applied to reduce the Ag NP size. In another study, Lee et al. [96] also used an AgNO_3_ containing solution but in a bipolar pulsed electrical discharge system with a needle to needle electrode geometry. In this case, the used surfactant to prevent aggregation of the fabricated Ag NPs was cetyltrimethylammonium bromide [CTAB; CH_3_(CH_2_)_15_N(CH_3_)_3_Br] and ultrapure water was used as solvent. Depending on the discharge time, Ag NPs with different sizes were produced. It was revealed that both Ag NP size and number increased with treatment time and with the concentration of silver nitrate in the solution. An addition of 30% or more surfactant in the solution was also found to be appropriate to prevent Ag NP aggregation. In another study, an atmospheric pressure helium (He) micro-plasma treatment was performed on an aqueous AgNO_3_ solution, with sucrose as the surfactant or stabilizing agent, to synthesize Ag NPs possessing diameters varying from 7 to 13 nm [97]. Finally, Cho et al. [74] employed a wire explosion process in liquid phase for Ag NP synthesis in both water and air media. The results illustrated that the size of the particles formed in water was smaller than the ones fabricated in air. The authors concluded that the high energy deposition in the water, the sufficient expansion volume and the quick cooling of the vapor are the main reasons for the creation of smaller particles in the water medium.

Besides silver and gold, there are also many experimental studies focusing on the synthesis of other metal NPs using various plasma discharges. For example, copper NPs have been produced using plasma electrochemical deposition in ionic liquids [98] and using a liquid phase bipolar pulsed plasma discharge in water [99]. The same bipolar pulsed discharge in water has also been used for the preparation of aluminum [73] and cobalt [100] NPs. A 2.45 GHz microwave-induced plasma in ethanol was also applied by Amaliyah et al. [101] to reduce ZnO powder in an effort to effectively produce Zn NPs. Synthesis of magnetic iron and cobalt carbide NPs encapsulated by a graphite shell was also studied by Sergiienko et al. [102,103] using an Ar DC discharge in an ultrasonic cavitation field, while Chen et al. [66] produced zirconium carbide NPs encapsulated in graphitic carbon using a pulsed plasma in ethanol to reduce a zirconium metal electrode. Finally, using an atmospheric pressure glow discharge, Toriyabe et al. [71] produced various metallic nanoballs (Ni, Ti, etc.) by plasma electrolysis.

**Figure 4:**
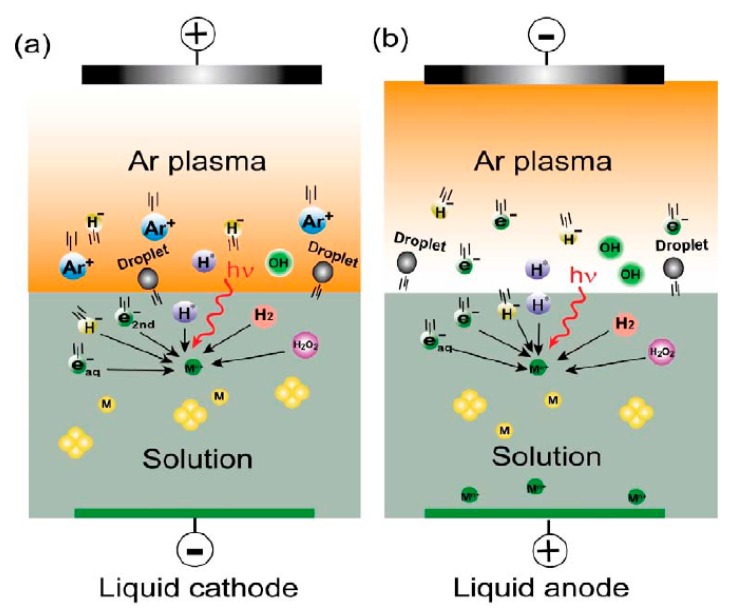
Liquid acts as (**a**) cathode and (**b**) anode. Reprinted with permission from [56].

Some well-designed reviews on the use of plasmas in the nanoscience field have already been published before. Graham et al. [106] reviewed a variety of electrical discharges employing electrodes immersed in the liquid for nanoscience applications. Additionally, Kareem and Kaliani [20] have presented a general review of glow discharge plasma electrolysis for NP synthesis. Recently, Saito et al. [107] summarized the available electrode configurations in plasma-liquid systems for nanomaterial synthesis in more detail. Finally, a well-documented review was very recently presented by Chen et al. [56] on the physical and chemical processes taking place during NP synthesis in plasma-liquid systems. Therefore, the readers are referred to the above-mentioned paper for more details on the processes occurring during plasma-assisted NP synthesis to avoid prolongation of this particular section. Nevertheless, the most notable information from the review paper of Chen et al. is summarized in this review paper and can be found hereafter:

- When the liquid acts as cathode in a plasma over liquid system, the voltage largely falls at the liquid surface. On the other hand, for the liquid-anode case, the voltage does not fall and electrons shower from the bulk plasma onto the liquid surface (see Figure 4) [56].
- In both the liquid-cathode and liquid-anode cases, the secondary electrons can dissolve into the liquid to form hydrated electrons (e_aq_^−^), which are very strong reducing species in the NP synthesis process [56]. Moreover, other plasma species including atomic hydrogen, H_2_, H_2_O_2_ (with different reducing abilities depending on the pH value) and hydride (H^−^) (very strong reducing species) are also able to reduce metal ions in the solutions in both cases. However, it has been reported that the yield of these reducing species in the liquid-anode case is much less than the yield in the liquid-cathode case. During PLIs, some oxidizing species can also be generated including atomic oxygen, ozone and OH^•^ radicals [56].
- The pH of the plasma-treated liquid plays an important role in the NP synthesis process. For example, the reduction ability of H_2_O_2_ is stronger in basic media than in acidic ones [56].
- “Plasma in liquid” systems show a relatively higher reducing efficiency compared to the “plasma over liquid” systems [56].
- Sputtering and evaporation are two physical processes frequently used for nanomaterial synthesis in plasma-liquid systems [56].
- It was shown that the type of metal ions strongly affects the synthesis process and also strongly affects the quality of the resultant NPs. For example, under similar plasma conditions, Au NPs were observed at both plasma cathode and anode, while Ag NPs were only found at the plasma cathode [56].

Although plasma-liquid systems are already successfully used for nanomaterial processing, NP synthesis and surface functionalization, further attempts must be made to optimize and extend the role of PLIs in this field by comprehensive understanding of the plasma-induced physical and chemical phenomena. Gathering this fundamental understanding can open new potential applications of PLIs in the field of nanomaterials. Although the complexity of plasma-liquid environments makes their understanding very challenging, new insights will continue to be reached by taking advantage of highly advanced imaging techniques and spectroscopic tools as well as by using multi-physics and chemistry simulations.

### 3.2. Plasma in Analytical Chemistry

**Figure 5:**
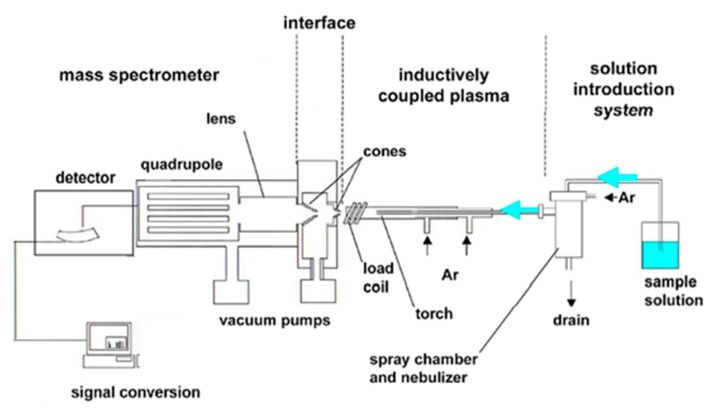
A typical inductively coupled plasma (ICP) system. Reprinted with permission from [116].

Plasma discharges are frequently used in analytical chemistry, for the analysis of gaseous, liquid or solid samples. More specifically, an inductively coupled plasma (ICP), which is produced by electromagnetic induction and which typically operates in Ar at atmospheric pressure is most commonly used, as shown in Figure 5. A liquid sample to be analyzed can be introduced into the plasma via several methods including thermal vaporization [108], electro-thermal vaporization [109,110], laser ablation [109] and spraying by means of a nebulizer [111,112,113]. The latter method, with a clear resemblance to spray discharge reactors, is applied when ICP is coupled to high precision liquid chromatography, for instance see [113,114]. Laser ablation of a liquid sample, on the other hand, has similarities to laser-induced breakdown, as the produced vapor plume has a sufficiently high temperature to form plasma. For reviews dealing with the state-of-the-art of these techniques for applications in environmental and life sciences, the interested reader is referred to [112,113,115].

**Figure 6:**
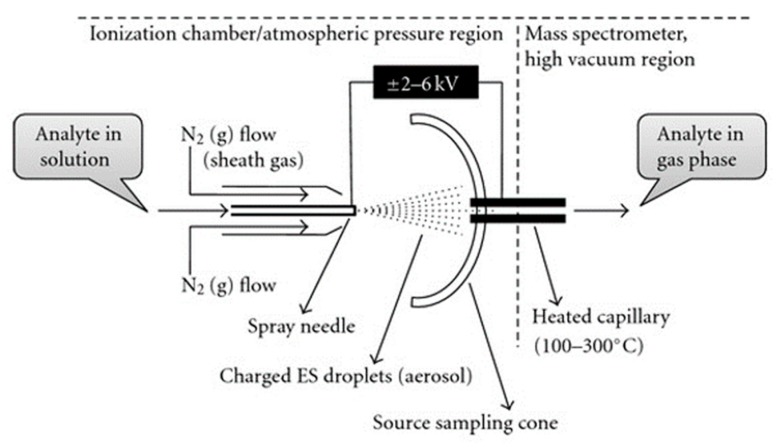
A schematic representation of the electrospray ionization ion source. Reprinted from [119].

A plasma discharge is present in various other established chemical analysis techniques as well, especially in the instrumental methods that include mass spectrometry or radiation spectroscopy. For mass spectrometry, different ionization methods are available to transfer the sample to the plasma state. One example is matrix-assisted laser desorption ionization (MALDI), where the analyte is mixed with a matrix and subsequently ablated by a laser pulse to form plasma. In classical MALDI methods, the matrix is crystalline, but the use of a liquid matrix has been shown to have advantages over a solid one [117,118]. A second example is electrospray ionization, where intermediate droplets are dispersed into the gas phase from a Taylor cone as an aerosol under influence of Coulomb repulsion (Figure 6). The charged droplets decrease in size through a combination of evaporation and fission processes, finally resulting in the formation of gas phase ions. As a third example, the analyte in a liquid matrix can be ionized by impact of high energy atoms or ions, which is referred to as fast atom bombardment or liquid secondary ion mass spectrometry, respectively. All three examples are evidently relevant in the context of PLIs.

Apart from laser-induced ablation as a preceding ionization method in combined hyphenated procedures, laser-induced breakdown spectroscopy (LIBS) has also emerged as a sufficiently mature independent analytical technique for the investigation of liquids, solids and gases. It stands out from other analytical methods in particular due to the absence of sample preparation and its capability to take measurements remotely from the sample. Additionally, there is a recent trend towards miniaturization and portability. Considering the versatility and recent advancements of LIBS, it has been proposed for application in environmental monitoring, in geochemistry, for microanalysis, for analysis of aerosols, bioaerosols and combustion, in benchtop instrumentation, in forensics, in conservation studies, for pathological use and toxicology assessment in biomedical fields, as sensor for sorting in recycling processes and as an inspection method for pharmaceuticals, industrial and nuclear processes [120]. The further improvement of LIBS is strongly dependent on the developments in fundamental knowledge of analyte sampling, vaporization and excitation, which are the physical processes that lie at its core. It should be noted, however, that the fundamental mechanisms in LIBS for liquid analysis are very different compared to solid or gas analysis. The state-of-the-art of LIBS is discussed extensively in [120,121]. Recently, several alternatives or modifications of the classical LIBS method have also been proposed to increase the detection sensitivity [122,123].

**Figure 7:**
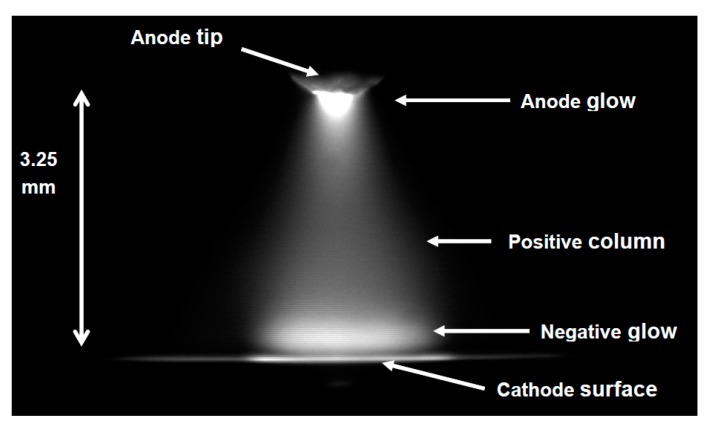
An intensified charge-coupled device (ICCD) camera picture of a typical ELCAD plasma operating between an electrolyte cathode and a tungsten anode. Reprinted with permission from [135].

Liquids, with water in particular, can also be analyzed by means of direct contact with electrically induced plasma. An electrical discharge in contact with liquids generally leads to mass transfer from the liquid towards the plasma phase, through processes such as evaporation and sputtering. Consequently, dissolved species are brought into the plasma, where they can undergo various processes including excitation and dissociation, associated with photon emission. Therefore, interpretation of the optical emission spectrum of the plasma can provide immediate information on the liquid content, which makes plasma an attractive tool for metal ion detection in water especially. Nonetheless, many non-thermal plasmas have a gas temperature that is too low for liquid evaporation [124]. For that reason, the method of sample introduction into the plasma plays a crucial role. In electrolyte-cathode discharge (ELCAD) spectroscopy, metal ions from an electrolytic solution, which serves as the cathode, are transferred to a contacting DC glow discharge by cathode sputtering (see Figure 7) [125]. Complete evaporation was achieved with a liquid sampling-atmospheric pressure glow discharge, which was sustained between a stainless steel capillary, used for delivery of the sample and a metal rod anode [126]. Also, AC powered discharge systems with an electrolytic solution as electrode [127] or AC powered DBDs over a liquid [128] can be used to reduce power consumption and liquid flow rates. In a drop-spark discharge, an electrolytic drop is atomized by electric breakdown during DC voltage application between the lowering drop and an electrolytic solution [129]. Glow discharges can similarly be generated between two liquid jets for liquid analysis [130]. Metal detection is also possible with electrohydraulic discharges including diaphragm discharges [131], capillary discharges [132], and electrical discharges at ultra-sharp tips and nanotubes submerged in the liquid [133]. Although the analytical performance of these techniques is well characterized, the underlying mechanisms through which the electrical discharge atomizes and excites the analyte remains poorly understood. A detailed recent review on aqueous metal detection by means of electrical discharges in direct contact with the analyzed solution can be found in [134].

### 3.3. Organic Wastewater Treatment

Water is one of the most important substances on the planet, with usage as a medium in multifarious industrial and natural processes. Depending on the process, a certain purity is required to employ the water solution while post-treatment aims to reduce or avoid pollution of the receiving environment after consumption. Therefore, water treatment is relevant for multiple water types such as drinking water, wastewater, seawater, and industrial medium. Water pollution is generally classified into debris, organic, inorganic, and biological waste [136], while additionally there is the upcoming issue of NP pollutants. In conventional water treatment plants, coarse debris and solids are removed during a primary treatment, where debris is filtered out by screens, heavy solids settle to the bottom and light solids, oil and grease float towards the surface. Although a part of the dissolved organic, inorganic and biological waste is separated as well throughout this process, their elimination is the main motivation for using a secondary treatment, by means of biological methods. Nonetheless, the removal efficiency for several specific persistent and hazardous pollutants up to this stage is found to be low. Hence, a tertiary treatment is often advised, in order to enhance the overall removal efficiency. Such a tertiary treatment can consist of chemical addition, advanced separation technology and/or advanced oxidation processes. Chlorination, while effective for biological decontamination, is insufficient for the removal of several bio-recalcitrant compounds. Advanced separation techniques such as coagulation-flocculation, micro- and nanofiltration, reverse osmosis, and activated carbon, have the disadvantages of high energy costs and the disposal of toxic residue concentrate. Advanced oxidation processes, on the other hand, are able to decompose toxic organic compounds and have therefore received increasing attention in recent years. Their implementation is, however, impractical up to now in most cases, due to their high energy demand and largely unknown side-effects. Accordingly, current research needs to focus on the enhancement of these methods, in order to press energy costs and to assure a low effluent toxicity. Ozonation is the most popular and developed oxidation method, based on more than a century of research. In principle, ozonation is a form of plasma treatment, since the employed ozone is generated by means of an electrical discharge before contact with the solution. Similarly, many UV-based methods apply plasma lamps including specialized excimer lamps, to decompose organic compounds. As plasma in not directly interacting with the liquid medium in these methods, a comprehensive discussion is out of the scope of this review. These methods are often combined with the addition of H_2_O_2_, to achieve higher decomposition efficiency. Unfortunately, this approach is complicated by the storage and handling of H_2_O_2_. Consequently, plasma treatment of water is an attractive alternative as it is able to generate a wide spectrum of reactive species such as ozone, H_2_O_2_, and UV photons, in proximity of the solution under treatment. Additionally, the wide variety in plasma reactors allows a flexible design and storage of chemicals is not required.

The present section further deals with plasma treatment of organic and inorganic water pollutants while the inactivation of aqueous biological targets such as bacteria and viruses by means of plasma discharges is covered in detail in Section 3.4. Decomposition of organic or inorganic compounds in water by plasma is believed to mainly arise through oxidation processes initiated by OH^•^ radicals, atomic oxygen O(^3^P), O_3_, and H_2_O_2_. In the gas phase, various types of reactive species can be formed, which transfer into the water with or without conversion into new reagents by interaction with the water molecules. Apart from that, electrolytic processes at submerged electrode surfaces and at the plasma-liquid interface can introduce new species as well. Next, the resulting aqueous species contribute to the plasma–chemical processes in water. Also, reductive reactions induced by H^•^ and superoxide anion (O_2_^•^^−^) radicals and molecular hydrogen can take place. The water solubility and oxidation potentials of some important reactive plasma-produced species are presented in Table 2. The majority of studies on water treatment by means of plasma discharges focuses on the decomposition of organic compounds. Goheen et al. [137] and Maximov and Kuzmin [138] investigated the destruction of organic compounds in water using a DC discharge burning between the water surface and an electrode. Radicals produced in the discharge react with air creating a gaseous mixture of ozone (O_3_), nitrogen oxides, and nitride acid aerosols that subsequently dissolve in water and react with the organic contaminants. Often, dyes are chosen as target pollutant, due to their hazardous impact on the ecosystem as waste from the textile industry and due to the wide availability of adsorption spectroscopy to determine their decomposition kinetics. Phenolic water pollutants are popular as well in oxidation studies using non-thermal plasmas, as summarized in two recent review papers by Zhang et al. [139] and Jiang et al. [140]. In recent years, increasing attention is also paid to many other types of organic compounds including pesticides, pharmaceuticals, and personal care products [141,142,143,144]. These compounds often manifest themselves in wastewater and natural waters in concentrations of the order of µg/L or lower and are accordingly referred to as “micropollutants”. Their toxicity, ecotoxicity, persistence in the environment, and resistance to removal by conventional wastewater treatment techniques make them compounds of emerging concern. Despite their occurrence in low concentrations, most studies on their degradation kinetics by plasma treatment apply these species as spiked pollutants in deionized water with concentrations well beyond 1 mg/L. Consequently, there is thus a clear need for future studies on the reaction kinetics in the case of more realistic concentrations and water matrices.

**Figure 8:**
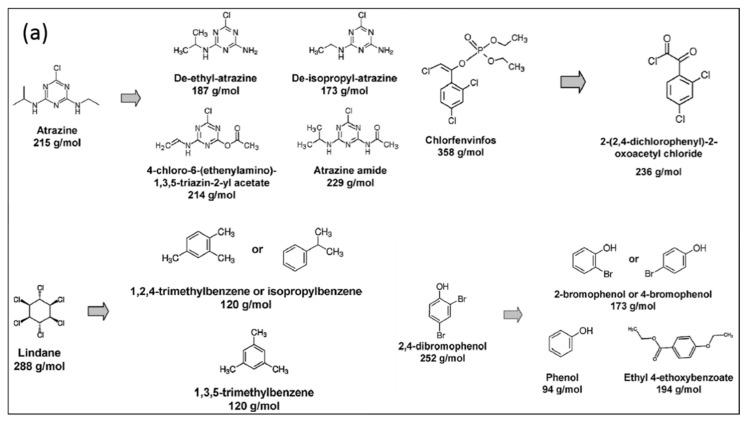

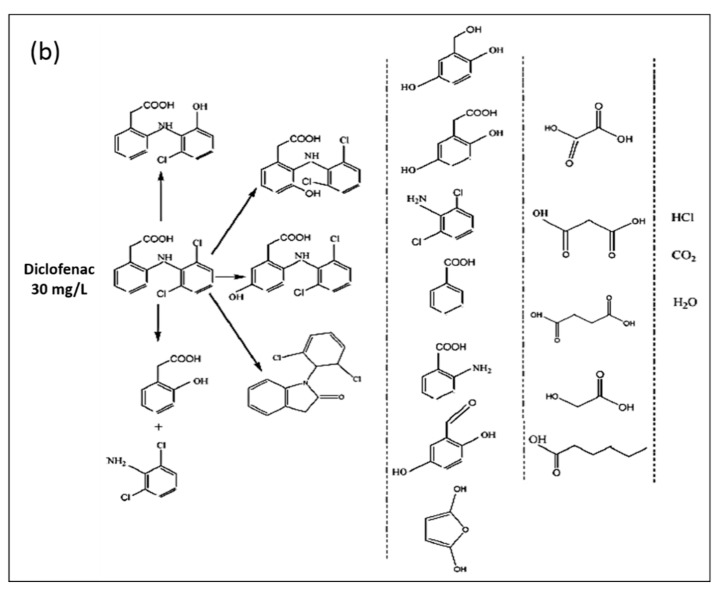
Proposed decomposition kinetics of Atrazine, Lindane, Chlorfenvinfos and 2,4-dibromophenol by Hijosa-Valsero et al. (reprinted with permission from [144]) (**a**) and proposed electro-oxidation degradation pathway of Diclofenac by Zhao et al. (reprinted with permission from [152]) (**b**).

Besides studying the decomposition kinetics of organic compounds, investigations of the oxidation pathways are frequently performed as well (see for example Figure 8) [149,150,151]. Due to the wide spectrum of plasma-generated reactive aqueous species, decomposition can occur in various oxidation steps including dealkylation, hydroxylation, addition of double bonded oxygen, nitrification, dehalogenation and, in case of aromatic compounds, cleavage of the aromatic rings (see a review by Magureanu et al. [142]). However, the amount of reports that focus on toxicity analysis of the plasma-treated solutions is very small while these are more relevant in the quest to bring this type of advanced oxidation technology closer to real-life applications. Namely, even when oxidation pathways are identified, it is seldom clear how toxic all oxidation by-products are in comparison to the parent compound. Moreover, besides the possible formation of hazardous organic and inorganic oxidation by-products, toxicity of the treated solutions can also increase due to transfer of toxic species from the gas phase into the liquid phase, especially in gas phase discharge reactors, or due to the formation of hazardous NPs by erosion of the electrode in electrohydraulic discharge reactors. Another challenge in electrohydraulic discharge reactors is the limitation posed by the electrical conductivity of the water to be treated on the production of pulsed electric discharges. At low electrical conductivity, below 10 µS/cm, the range of the applied voltage required for the production of the corona discharge without sparking is narrow. On the other hand, at high electrical conductivity (above 400 µS/cm), which is the typical conductivity of tap water, streamers become short and the efficiency of radical production decreases [146]. Generally, OH^•^ and atomic oxygen O(^3^P) can be produced more efficiently at water conductivity values below 100 µS/cm. Hence, this is one of the major challenges in the plasma treatment of water as the electrical conductivity of most occurring water lies in the range 2000–4000 µS/cm. Another challenge is related to the treatment of seawater, for which the conductivity can even be higher than 30,000 µS/cm [146].

**Figure 9:**
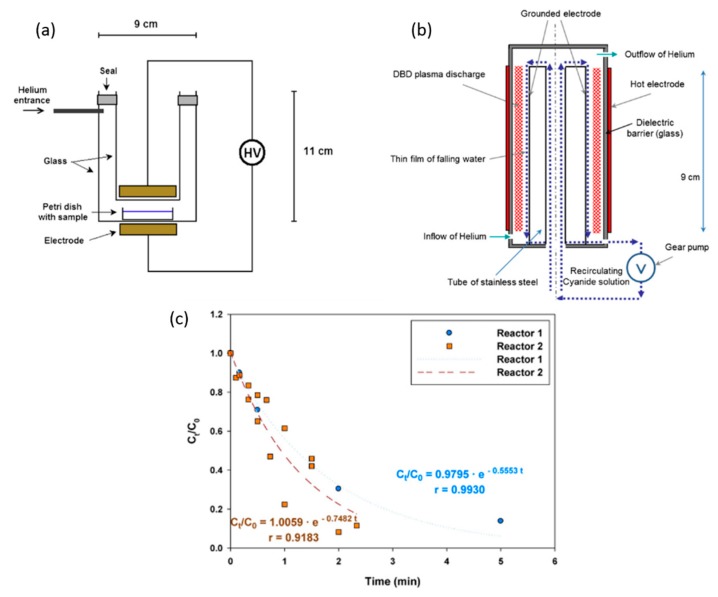
(**a**) DBD batch reactor (Reactor 1) and (**b**) coaxial thin film DBD reactor (Reactor 2) used by Hijosa-Valsero et al. [154] to remove cyanide from distilled water; (**c**) removal of cyanide from water in both reactors (initial concentration of cyanide: 1 mg/L). Reprinted with permission from [154].

Besides the removal of organic compounds, plasma discharges can also be used for the elimination of inorganic species. Oxidation of I^−^, Br^−^, S^2^^−^, Cr^2+^ and Mn^2+^ ions by steady-state discharges between a metal anode and an electrolyte was investigated in [153]. More recently, Hijosa-Valsero et al. [154] have reported cyanide removal by means of two different DBD reactors shown in Figure 9. Although it is well known that PLIs, as other advanced oxidation processes, can be applied for the oxidation of inorganic contaminants, the amount of reports that focus on this application is scarce. Previously, it was already mentioned that reactive species with higher oxidation potential tend to be short-living. Consequently, more studies are also needed to understand how these short-living reactive oxidative species can be made to work more effectively.

At this moment, for real-life applications in water treatment, plasma treatment is a priori disfavored as compared to other advanced oxidation processes, as it is looked upon as a more complex technology than its competitors. Therefore, it needs to perform significantly better than any competitive technology, instead of as good, in order to be implemented on a large scale. To reach this high level of performance, more comparative studies are required to gain a clear overview on the current state of affairs with regard to energy efficiency of the different technologies. Additionally, up to now, studies on water purification by means of advanced oxidation processes in general and plasma treatment in particular have mainly focused on reactor effectiveness and energy efficiency for degradation of single compounds. Although this approach allows researchers to compare different reactors and techniques, it gives insufficient information on the quality of the solution after treatment. In order to gain this information, detailed reports on reactor optimization in terms of effluent toxicity reduction are needed. However, such reports, if any, are sparse for most advanced oxidation techniques. As a direct implication, a new line of research is required with a stronger focus on overall toxicity and post-treatment effects, if this young technology is to be brought closer towards real-life applications.

### 3.4. Plasma Sterilization and Disinfection

Although the term plasma sterilization of water is often used, the technique is not really a sterilization process as it is not able to completely destroy harmful or pathogenic living microorganisms in water. Actually, plasmas can only reduce the number of harmful microorganisms to levels appropriate for the intended use of the receiving water [155]. The first employment of plasmas for biological applications dates back to the late 1850s, when a DBD was used by Siemens to generate ozone for cleaning water containing biological contaminants [156]. A few attempts to use plasmas for water sterilization were also made from the 1960s to the 1980s [157]. In the early 1980s, there was a small revival of the use of microwave (MW) plasmas with biocidal characteristics for water sterilization purposes [158]. However, it took more than 130 years after Siemens, in the mid-1990s, before researchers started to conduct research with the aim to understand the interactions between plasma and biological cells. During the same period, atmospheric non-thermal plasmas also became a very hot research topic for scientists and some of them started to investigate the effects of these plasmas on bacterial cells [159,160,161,162,163,164,165].

At this moment, many plasma-based sterilization studies have been carried out to inactivate microorganisms under dry conditions [166,167,168,169]. However, for multiple practical applications, sterilization under wet environments is also important. In a wet environment or in a gel-like material with sufficient water content, the situation is different from the one in a dry state and possibly much more complex. Microorganisms have much better living and growth conditions in wet state, while a wet environment also gives an additional barrier, which will impede direct plasma–microorganism interactions. As a result, plasma-induced effects on microorganisms will be strongly affected by the presence of a liquid phase. Various studies have already shown that exposure to a non-thermal atmospheric pressure plasma can inactivate different bacteria suspended in a liquid, of which numerous examples can be found in Table 3. Within this context, conversion of the applied electrical energy into chemical species during PLIs is of specific interest as radicals and molecules formed in the liquid phase can initiate a wide variety of chemical and biochemical reactions. The microorganisms can be effectively inactivated (and organic contaminations can be oxidized) as a result of contact with reactive plasma species and radicals. Although a multitude of species can be generated in the liquid phase due to plasma exposure, hydroxyl radicals OH^•^, atomic oxygen O(^3^P), ozone O_3_, and hydrogen peroxide H_2_O_2_ are considered to be the most important ones for sterilization [146]. In addition to these chemical reactive species, some physical processes including UV photolysis, large electric fields, and shock waves can contribute as well to the bacterial inactivation processes. For this, UV/VUV radiation generated in the plasma gas phase has to penetrate or diffuse into the liquid to be able to interact with microorganism cells. Moreover, plasma-induced changes of the liquid environment itself may also contribute to biological plasma effects. UV radiation in the wavelength range of 280–240 nm UVC (Table 4) was claimed to possibly lead to irreversible damage to the nucleic acid of microorganisms, preventing proper cellular procreation and therefore effectively inactivating the microorganisms [146]. Laroussi et al. [170] also suggested that UV radiation may produce charged particles in water in such a way that charge accumulation occurs on the outer surface of the membrane of a bacterium cell. Subsequently, the generated electrostatic force on the membrane overcomes the tensile strength of the cell membrane, causing its rupture at a point of small local curvature as the electrostatic force is inversely proportional to the local radius squared. Additionally, also photons can be responsible for providing the necessary ionization or dissociation energy of water molecules, generating reactive chemical particles. Finally, shock waves, produced by the plasma during PLIs, can also significantly enhance the plasma treatment efficiency in sterilization processes as they help to mix the liquid [171,172].

For a long time, it was believed that inactivation of bacteria in aqueous liquids by atmospheric pressure plasma treatments is highly dependant on acidification as acidification is one of the most notable chemical changes that plasmas induce in liquids. However, in the last decade, it was demonstrated that acidification alone does not induce a comparable bactericidal efficacy [173,174,175]. Consequently, researchers concluded that bacterial inactivation is more likely based on the diffusion of plasma-generated species through cell membranes into the cells where they react and possibly damage proteins and nucleic acids, leading to cell death [176,177,178,179]. Additionally, the plasma-induced acidification itself was attributed to the multistep reactions occurring between plasma reactive species generated in the gas phase and the liquid at the plasma-liquid interface. For example, it was suggested that acidification by plasma exposure is caused by the dissolution of nitrogen oxides generated in the gas phase inside liquids [180]. Besides acidification, plasma treatment of aqueous liquids also results in other changes to the liquid caused by the generation of reactive species such as H_2_O_2_, O_3_, atomic oxygen, NO_x_ and so on, which are known to have oxidizing effects.

Due to the acidic effects of plasma treatment of liquids, some groups have tried to reveal which acids are specifically created in plasma-treated liquids and which chemical reactions play dominating or supporting roles for acidification and subsequent antimicrobial activity. Shainsky et al. [179] examined two different hypotheses; the first one was nitric/nitrous acid formation and the second one was the formation of an acid, they called “plasma acid”, containing hydrogen cations (H^+^) and O_2_^•^^−^ radicals. Although some groups [181] introduced nitric/nitrous acid as a possible source of acidity and oxidative ability of plasma-treated water, Shainsky et al. observed that pH reduction in plasma-treated liquids takes place not only in air but also in oxygen. Based on these observations, they mentioned that nitric acid formation cannot explain the acidity of plasma-treated water in the presence of oxygen. Moreover, the intensity of the UV absorption peak at 300 nm, specific for nitric acid, was reported to be significantly lower for the O_2_ plasma-treated water than in case of the air plasma-treated samples. As such, they proposed a second possibility (the presence of “plasma acid”) to explain the observed acidic effects of plasma treatment. They also suggested that the strong oxidizing properties of peroxides such as H_2_O_2_ and O_2_^•−^, present in the pulsed DBD plasma-treated water together with the acidic environment resulted in a strong oxidizing solution, effective for inactivation of *E. coli* bacteria in deionized water. In another study, Satoh et al. [177] used air, O_2_, and N_2_ pulsed plasmas generated between a multi-needle electrode and water containing *E. coli* for disinfection. In the case of air and N_2_ plasmas, a decrease in pH from 7.3 to values between 3 and 4 was observed due to the dissolution of nitrogen oxides (NO_x_) produced in the gas phase in the water. However, no acidification was detected when applying the O_2_ plasma treatment. Consequently, the authors concluded that acidification of the water may contribute to the decay of the *E. coli* density in the case of air and N_2_ plasma exposures. On the other hand, a higher bacteria inactivation activity was reported when applying the O_2_ plasma where no acidification was observed. This observation led the researchers to the conclusion that the bactericidal action in case of O_2_ plasma is likely due to the dissolution of O_3_ produced in the plasma because a high concentration of ozone was obtained only when O_2_ was used as discharge gas.

In contrast to the work of Shainsky et al. [179], nitric acid formation from plasma-generated reactive nitrogen species (RNS) was considered to be the main source of liquid acidification by Oehmigen et al. [173]. However, a strongly increased H_2_O_2_ concentration was also observed after both indirect DBD plasma treatment and only NO gas treatment. Consequently, they concluded that reactive oxygen species (ROS) must have more strength for subsequent microbial inactivation. Woedtke et al. [175] also reported that low molecular nitrogen and oxygen containing chemical species should play a key role in both acidification and microbial inactivation. The short-term increase of nitrite and its relatively low concentration compared to nitrate lead them to conclude that under acidic conditions, a disproportionation of HNO_2_ into HNO_3_ and nitric oxide (NO^•^) takes place which is generally accelerated by heating and a concentration increase:

(1) $$3{\mathrm{HNO}}_{2}\to {\mathrm{HNO}}_{3}+2{\mathrm{NO}}^{•}+H_{2}O$$

Their first assumption was that HNO_3_ is the main cause for acidification, because they did not observe a temperature increase resulting from plasma treatment. Therefore, due to the strong acidity of HNO_3_ (pK_a_ = −1.3) and its continuously increasing concentration, a continuously decreasing pH dependent on plasma treatment time was expected. However, their measurements resulted in a stabilization of the pH value around 3. Consequently, it was assumed that not HNO_3_, but HNO_2_ is playing the lead in acidification, because its pK_a_ value lies between 3.2 and 2.8 and HNO_2_ showed temporarily increasing concentration with treatment time. The UV-vis results obtained by the authors also indicated a predominating role of HNO_2_ in the acidification process when using an atmospheric air environment. However, the obtained results from Ar plasma treatment revealed that acidification even occurs in nitrogen-free discharge gases. Therefore, it should be noticed that nitrogen containing chemical species are not the only reaction channels, leading to acid generation in plasma-treated liquids.

*E. coli* in water and saline solutions was also treated using a DC transient spark discharge in air by Machala et al. [178]. These authors correlated the bactericidal effects with the formed peroxynitrites as well as with the oxidative stress induced in cell membranes. In their experiments, the concentrations of peroxynitrites were however too low compared to the measured nitrites and H_2_O_2_ concentrations to consider peroxynitrites as the key bactericidal agent. They found that the synergistic effects of nitrites and peroxides in acidic conditions are the most responsible factors for bactericidal properties. Ozone and iron NPs sputtered from the electrodes during the experiments were also considered as potential bactericidal agents. Some free radicals such as OH^•^ and the superoxide anion O_2_^•−^ have also been reported to contribute to bacterial inactivation in aqueous solutions [182]. However, the very short lifetime of OH^•^ radicals (200 µs at 1.0 × 10^−6^ M) and the relatively long lifetime of O_2_^•−^ radicals (5 s at 1.0 × 10^−6^ M) in aqueous solutions suggest that sufficient diffusion of the latter radicals in the solution for inactivation of microorganisms is more considerable [183,184]. However, although O_2_^•−^ radicals are reactive, they are considered to be incapable of penetrating the cell membrane due to their charges. Therefore, they are unlikely to cause DNA damages of bacterial cells. Korshunov et al. [185] however suggested that when the pH of a solution is sufficiently low, O_2_^•−^ radicals can convert to hydroperoxy radicals (HOO^•^) which can penetrate the cell membrane and damage intercellular components. The equilibrium reaction between superoxides and hydroperoxy radicals is as following:

(2) $$O_{2}^{•-}+H^{+}↔{\mathrm{HOO}}^{•}\;\;\;\;{\mathrm{pK}}_{a}\approx 4.8$$

Ikawa et al. [180] developed an atmospheric pressure He plasma jet where its UV or heat production is harmless to living cells and applied this plasma to the surface of an aqueous solution containing *E. coli* or *L. citreum* bacterial suspensions. They claimed that neither UV light nor heat from the plasma was the cause of bacterial inactivation. These authors also suggested the importance of the highly reactive species (e.g., hydroperoxy radicals (HOO^•^) and specially O_2_^•−^ radicals) generated in the solution via PLIs on the observed bactericidal effects. Their experiments also indicated that the combination of He with O_2_ (instead of air) results in weaker bacterial inactivation. This result suggests that the presence of nitrogen in the gas phase may also be necessary to account for a full bactericidal effect. One of the most interesting findings in their study is the introduction of a critical pH value (≈4.7) of the solution for bactericidal effects, below which the bacteria were sufficiently inactivated and above which the bacteria were hardly affected by the plasma. They suggested that this critical pH represents a strong association of O_2_^•−^ radicals with the bactericidal effects as the critical pH value is almost equal to the pK_a_ of the equilibrium reaction between O_2_^•−^ and HOO^•^ (~4.8). The authors also reported the same hypothesis as Korshunov et al. [185] for the observed bactericidal effects. Liu et al. [186] used an atmospheric DC air micro-jet inside water to inactivate S. aureus and a rapid change in the inactivation rate from 10% to 100% was observed when the pH value decreased to ~4.5, which is very close to Ikawa et al.’s critical pH value [180]. This inactivation was related to a change in the acidity of the liquid in conjunction with the direct interaction of plasma-activated species with the cells which initiate the peroxidation of the fatty acid in the cell membrane, resulting in the inactivation of the bacteria in an aqueous environment. The authors also proposed that O_2_^•−^ and its direct conjugate HOO^•^ play an important role in the initiation of the oxidation of the fatty acid in the cell membrane. Like Ilawa et al. [180], they also mentioned that the critical pH value is associated with the pK_a_ of HOO^•^ in aqueous solutions (Equation (2)).

Bai et al. [187] used a similar plasma jet as Liu et al. [186] in He/O_2_ for the inactivation of *S. aureus* in water. However, these authors suggested that the inactivation is mainly attributed to OH^•^ radicals generated in the water by the plasma exposure. The existence of OH^•^ radicals in their system was verified via electron paramagnetic resonance (EPR) spectroscopy. Other ROS such as O_2_^•−^, O_3_, and O(^3^P) were also found to exist in their system. They stated that because of their lower concentrations, other ROS besides OH^•^ radicals are suspected to be the precursors of OH^•^ radicals. For example, atomic oxygen can directly oxidize bacteria within its close vicinity or react with a water molecule first, generating intermediate species such as OH^•^ or H_2_O_2_ for the inactivation of bacteria. Additionally, the authors mentioned that trace amounts of ozone (a few ppm), detected in air, can live for 1000 s at room temperature when submerged in water. Consequently, it was believed that ozone can first interact with water to produce OH^•^ radicals and can therefore indirectly participate in the inactivation process of bacteria. Shen et al. [188] also performed similar experiments as Bai et al. [187] but used an atmospheric pressure DC bubbling discharge in Ar. They also mentioned that OH^•^ radicals are supposed to play an effective role in the plasma inactivation process, but together with atomic oxygen O(^3^P) radicals.

Both nitric and nitrous acids are also proposed by Kojtari et al. [189] as the main possible chemicals that contribute to acidification of plasma-treated liquids. However, unlike Woedtke et al. [175] who assumed that HNO_2_ is the main cause for acidification, they concluded that the major acid in the solution that contributed to the low pH value is HNO_3_ and ruled out a major contribution from other acids such as hydroperoxyl (HO_2_) and peroxynitrous acid (ONOOH). They also mentioned that HNO_2_ is not the major component for the observed antibacterial activity in DBD plasma-treated water due to its high instability. Very low concentrations of H_2_O_2_ also led to the conclusion that this chemical species cannot be responsible for complete inactivation of *E. coli*. The authors followed Ikawa et al.’s [180] hypothesis to answer the question which chemicals in the solution play a major role in the antimicrobial properties of the plasma-treated water. However, unlike Ikawa et al. [180], the Raman spectroscopy and mass spectroscopy results they obtained made the authors believe that HOO^•^ cannot be responsible for the antimicrobial property of the liquid because of its very low concentration which was caused by very rapid reactions between HOO^•^ and radicals. The authors also considered ONOOH as a potential antimicrobial agent due to its strong oxidation ability and relatively long lifetime, but because of its instability in acidic conditions and its very low concentration, they were not able to detect it with Raman spectroscopy. However, existence of ONOOH in the treated solution was confirmed by EPR and they stated that “it is generally believed that peroxynitrite is stable only in alkaline conditions, however, based on all the results we observed, we tend to believe that peroxynitrite is stable in the unique low pH environment generated in the plasma-treated water and it is likely the origin of the antimicrobial property of the plasma-treated water. The reason should be further studied in the future.”

**Figure 10:**
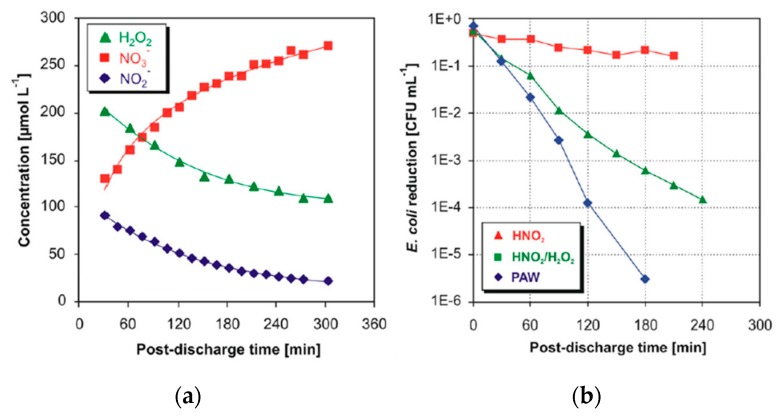
(**a**) Post-discharge evolution of three main plasma-generated chemical species in aqueous solution (pH = 3.3) and (**b**) comparison of inactivation of *E. coli* in three different solutions: HNO_2_ (nitrites only), HNO_2_/H_2_O_2_ (mixture of nitrites and H_2_O_2_), and PAW (plasma-activated water). Reprinted with permission from [190].

Lukes et al. [190] applied a pulsed discharge plasma in O_2_/N_2_ and O_2_/Ar to an *E. coli*/water solution and mentioned that in the case of the O_2_/N_2_ plasma, the bactericidal effect involves synergistic effects of nitrites and peroxides in acidic conditions through the cytotoxic activity of the produced secondary species. The actual concentration of H_2_O_2_, NO_3_^−^ and NO_2_^−^ was observed to be significantly dependent on the pH of the air plasma-treated water. They reported a lower concentration of NO_2_^−^ and higher concentrations of H_2_O_2_ and NO_3_^−^ in acidic conditions. The authors also studied post-discharge reactions occurring in the liquid phase. As shown in Figure 10, as the concentrations of H_2_O_2_ and NO_2_^−^ decreased with post-discharge duration accompanied by an increase in concentration of NO_3_^−^, they proposed that HNO_2_ can rapidly decomposes into NO and NO_2_^•^ because of the instability of NO_2_^−^ under acidic conditions. Also, the formation of NO_3_^−^ may proceed through the reaction of NO_2_^−^ with H_2_O_2_ to form peroxynitrous acid ONOOH:

(3) $${\mathrm{NO}}_{2}{}^{-}+H_{2}O_{2}+H^{+}\to O=\mathrm{NOOH}+H_{2}O$$

(4) $$O=\mathrm{NOOH}↔{\mathrm{OH}}^{•}+{\mathrm{NO}}_{2}^{•}$$

In the case of O_2_/Ar plasma, peroxone chemistry of ozone with hydrogen peroxide was proposed to enhance the bactericidal effects with increasing pH. The authors hypothesized that ozone from the plasma can be dissolved into the plasma-treated water and decomposes under alkaline conditions via a series of chain reactions to produce secondary OH^•^ radicals in water:

(5) $$2O_{3}+H_{2}O+{\mathrm{OH}}^{-}\to {\mathrm{OH}}^{•}+{\mathrm{HO}}_{2}^{•}+2O_{2}+{\mathrm{OH}}^{-}$$

The presence of H_2_O_2_ accelerates the decomposition of ozone and increases the OH^•^ radical concentration:

(6) $$O_{3}+H_{2}O_{2}+{\mathrm{OH}}^{-}\to {\mathrm{OH}}^{•}+{\mathrm{HO}}_{2}^{•}+O_{2}+{\mathrm{OH}}^{-}$$

The authors mentioned that at low pH values, this process occurs very slowly, but at pH values above 5, the process is greatly accelerated. The overall bactericidal effect of the air plasma was associated with the effects of NO_2_^−^ and peroxides in acidic conditions through activity of secondary reactive species such as NO, NO_2_^•^, OH^•^, and ONOOH. However, the authors suggested that alkaline conditions (pH values above 5) are more suitable for bactericidal activity in the case of Ar/O_2_ atmosphere instead of air.

In a study by Gils et al. [191], it was indicated that the acidity of the treated solutions has a significant influence on the bactericidal effect because it was the pH level which determined the equilibria of various active chemical species such as peroxynitrite/peroxynitrous acid and nitrous acid but also determined the solubility of ozone which is known to play a key role in the antibacterial process. Gils et al. employed an atmospheric radiofrequency (RF) plasma jet in Ar to inactivate *P. aeruginosa* in water and saline. The authors believed that the bactericidal effect was partly related to the toxicity of the HNO_2_ acid. The effect of the liquid acidity on the bactericidal inactivation was explained by the correlation between the decreased pH and the increased concentration of nitrous acid. They also stated that the strong bactericidal effect of plasma-treated solutions can be a synergistic result of different plasma species. Additionally, it was also revealed from their results that RNS rather than ROS (with the exception of H_2_O_2_) play a more essential role in bacterial inactivation.

Takamatsu et al. [192] employed an AC multi gas plasma jet in CO_2_, N_2_, Ar, O_2_, and mock air to inactivate different bacteria (*S. aureus*, *E. faecalis*, *E. coli*, *P. aeruginosa*, MRSA, *M. terrae*, *M. abscessus*, *B. cereus*, *C. albicans*, *T. mentagrophytes* and *A. niger*) in phosphate buffered saline (PBS). Singlet oxygen and OH^•^ radicals were supposed to have the greatest inactivation effects. The authors observed that the largest amount of OH^•^ and singlet oxygen radicals were generated in the N_2_ and CO_2_ plasmas, respectively. However, because of the best bacterial inactivation which was obtained when using the N_2_ plasma, they suggested that probably the OH^•^ radical is more effective for bacterial inactivation than singlet oxygen radicals.

Park et al. [172] used two types of plasma sources: A nano-pulsed plasma (NPP) generated in the liquid and an Ar DBD plasma generated above the liquid to inactivate S. aureus in saline. Using both systems, they found that both ROS and RNS play a significant role in bacterial inactivation. Although more ROS/RNS were observed for the DBD plasma, the inactivation rate for the NPP plasma was greater than for the DBD. Based on scanning electron microscopy (SEM) results, they explained these results by a higher shock waves production in the NPP system than in the DBD which can crush many bacterial spores. The authors also did not believe that O_2_^•−^ was a major contributor to the bacterial inactivation process because it is converted to HO_2_^•^ at low pH and also because most cells contain superoxide dismutase that convert O_2_^•−^ to O_2_ and H_2_O_2_. Consequently, they suggested that HO_2_^•^ can play an important role in bacteria inactivation because of its ability to penetrate the cell membrane. Also, the reaction between NO and O_2_^•−^ was considered in their hypothesis as the second factor affecting the bacterial inactivation because this reaction leads to the production of a toxic and powerful oxidant, ONOO^−^. They also stated that the role of the pH is only to increase the reactivity of the radicals as the pH has no direct effect on the bacterial inactivation in their studies. The authors suggested that in an acidic solution, ONOO^−^ is protonated to form ONOOH which is a very strong oxidant for biomolecules and thus plays an important role in the antibacterial activity. Finally, they also suggested that shock waves play an important role in the inactivation process.

Contrary to all previous studies, Pavlovich et al. [193] concluded that the antimicrobial rates with ozone were much faster than previously reported antimicrobial rates for inactivation affected by nitrogen oxides and low pH values. The authors inactivated *E. coli* in saline and PBS and observed a good correlation between the antimicrobial effect and the liquid phase ozone concentration, but interestingly no correlation was observed between the antimicrobial effect and pH or concentration of H_2_O_2_, nitrite or nitrate. Based on these results, the authors suggested an ozone-dominant mechanism of *E. coli* inactivation and added that ozone-mediated inactivation is pH-independent under indirect air DBD plasma treatment. Further support for their hypothesis came from the measured liquid phase ozone concentration which was consistent with the estimated equilibrium ozone concentration in the gas phase following vigorous mixing of the phases.

Ercan et al. [194] examined *E. coli* inactivation in N-acetylcysteine (NAC) using an atmospheric air DBD plasma. They stated that pH has no direct significant contribution to bacterial inactivation, however, acidic pH was suggested to be crucial because of the antimicrobial effect of acidified nitrites. They also mentioned that although both ROS and RNS can attribute to the antimicrobial effect, it is speculated that ROS play an important role in the modification of NAC molecules while RNS seems to contribute more dominantly to the antimicrobial effect.

Even though some reports are available in literature which believe UV radiation [195,202] and ROS including ozone [177,193], hydroxyl radicals [187,188,192], singlet oxygen [192], and atomic oxygen [188] have more dominant bactericidal effects in plasma-based sterilization processes, a more prominent picture of the effects of RNS in plasma sterilization is observed in literature. Also, from these results, it can be concluded that acidification seems to be a necessary, but not sufficient prerequisite for antimicrobial effects. Many investigations have been performed up to date in an effort to obtain a deep understanding on which chemicals affect the bacterial inactivation and how these affect the sterilization efficacy during PLIs. However, the chemical kinetics of the reactions occurring between the plasma-generated chemicals and liquid phase components, remains an area of significant research. The production of these plasma-generated chemicals is depending on many parameters such as plasma environment, type of carrier gas, type of discharge, applied voltage, voltage polarity, the liquid volume, the distance between electrode and liquid surface, the electrical conductivity of the liquid, pulse duration and so on. It has been already shown that in lower electrode gaps a better inactivation efficacy was achieved. This observation can be due to the different discharge regimes and their different energy efficiencies which can be obtained when using varying electrode distances. Vaze et al. [195] observed a corona discharge at long electrode distance (5 cm), a corona-spark discharge at a 2.5 cm electrode gap, and a spark discharge at a 1 cm gap. They reported a more significant bacterial inactivation rate with a spark discharge compared to the other two discharge types. Later on, this group also investigated the effect of a gliding arc discharge over water [202] and observed that the spark discharge still had a higher efficiency than the gliding arc discharge over the water surface and the corona discharge. Additionally, the initial concentration of the bacterial solution was also found to have an important effect on the bacterial inactivation efficiency as it is more difficult to inactivate bacteria when they are present in high concentrations. Generally, a detailed detection of reactive species in the plasma- treated liquid phase is one of the essential forthcoming tasks to get further insights into the detailed mechanisms of PLIs on bacterial inactivation. For this, highly sophisticated liquid analytics need to be applied to plasma-treated aqueous solutions in the near future.

### 3.5. Plasma Biomedicine, Wound Healing and Blood Coagulation, Plasma Dermatology and Dentistry

Atmospheric non-thermal plasmas are currently also attracting significant attention in medical sciences, where for some applications the delivery of reactive oxygen and nitrogen species (RONS) is highly desired. Historically, in 1914, Jacques-Arsene d’Arsonval, a French physiologist, discovered the possibility of influencing the human body with high frequency irradiations [203]. Therefore, electro-medicine using high frequencies was recommended for medical practice in the first decades of the 20th century. The high frequency devices were commonly sold for home-care medicine until the early 1950s [204] and in Germany, these devices were further developed for diathermy [205]. In 1927, Rumpf designed a device different from the one of d’Arsonval, by implementing a capacitively coupled electrode system consisting of a Leydener bottle as an electrode, which was directly applied to the skin. His device can be considered as the first plasma source in biomedicine using a dielectric electrode [204]. Subsequently, more efforts took place using plasmas and in the early 1990s, and research was performed by Gitomer and Jones [206] at the Los Alamos National Laboratory to study laser-induced plasmas for medical applications including ophthalmology, urology, and cardiology. However, before 1998, only a few research groups were focusing on the plasma biomedicine field because many people in the plasma scientific community were not aware of this new application field of plasmas. However, in 1998, for the first time, after many efforts were made by Laroussi and Barker, first results of a coordinated efforts to investigate plasma–cell interactions were effectively presented [157].

Due to their low operating temperatures, non-thermal plasmas are suitable for the treatment of heat-sensitive and/or vulnerable materials such as living biological cells and tissues. Plasma-generated RONS exhibit high biological activity (e.g., anti-microbial, anti-cancer, and chronic wound healing) and are of critical importance in the biomedicine field because they can directly and indirectly affect plasma-induced chemical reactions on cells and tissues. Non-thermal plasmas typically produce a complex mixture of RONS including hydrogen peroxide (H_2_O_2_), nitric oxide, hydroxyl radicals (OH^•^), superoxide and ozone (O_3_), among others. H_2_O_2_ is considered as one of the key components in wound healing, anti-microbial, and anti-cancer properties of non-thermal plasmas [207]. The most straightforward pathways, leading to the formation of H_2_O_2_, are listed in Table 5. Also, a well-documented review was presented by Locke et al. [58] clearly summarizing H_2_O_2_ formation methods. Very short-living OH^•^ radicals, as an important precursor of H_2_O_2_, are also considered in biological applications [207].

**Figure 11:**
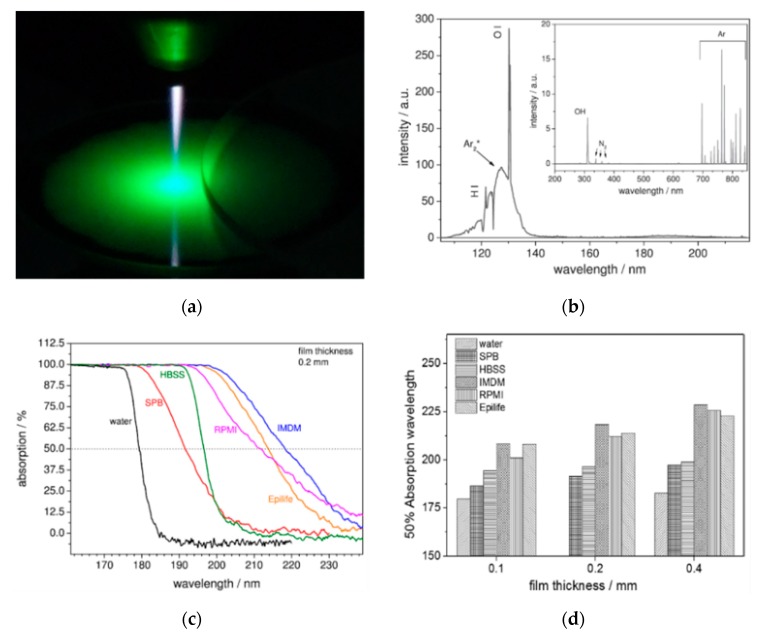
(**a**) VUV emission profile of an Ar plasma jet in a phosphorescing film used by Jablonowski et al., (**b**) VUV emission of an Ar discharge in ambient air, (**c**) VUV absorption of various test liquids at 0.2 mm sheath thickness and (**d**) wavelength at which 50% absorption of plasma-radiated VUV occurs of various test liquids as a function of their film thickness. Reprinted with permission from [208].

Besides RONS, UV radiation of the plasma was also found to be effective in biomedicine. The effects of UV (100–400 nm) and VUV (10–200 nm) radiation generated by an RF atmospheric pressure Ar plasma jet on different biological solutions were investigated by Jablonowski et al. [208] (Figure 11). The authors exposed the following liquids to their plasma jet: distilled water, 0.9% NaCl, Hank’s balanced salt solution (HBSS), Sørensen’s phosphate buffer (SPB), Dulbecco’s phosphate buffered saline (DPBS), Iscove’s modified Dulbecco’s medium (IMDM), EpiLife, and Roswell Park Memorial Institute (RPMI), respectively, sorted by complexity from ultrapure water to full culture media. The main aim of their work was to identify the possible impact of VUV radiation on liquids surrounding cells and based on the bond dissociation energies shown in Table 6, the authors concluded that VUV photons generated by their plasma jet carry enough energy onto treated liquids to contribute to the reactive chemistry initiated within the liquids. Additionally, the authors also studied the generation of H_2_O_2_ and reactive oxygen radicals namely O_2_^•−^ and OH^•^ radicals by the use of EPR spectroscopy inside the liquids.

In some early biomedical applications including tissue removal, sterilization and cauterization, heat and high temperature are also important. Consequently, atmospheric thermal plasmas have been also employed for biomedical applications such as cutting tissue, cosmetic re-structuring of tissue, and cessation of bleeding [211]. However, atmospheric non-thermal plasmas have a more interesting and promising role in biomedicine because their effects can be tuned for various purposes including prevention of post-surgical infections, non-inflammatory tissue regeneration, oncology, chronic wound healing, treatment of skin diseases, cancer therapy, dental caries, etc. Most efforts in plasma biomedicine are focused on applying plasma directly to samples including tissues, biological liquids and biomaterial surfaces. Therefore, using non-thermal plasmas helps to achieve the desired result selectively for some living matter while having little effect on the surrounding tissue [211]. The applications of plasma in medicine usually involve contact between plasma and tissue which requires a liquid surrounding, for example cell culture medium for in vitro studies. Also, under in vivo conditions, a liquid environment nearly always surrounds living tissue due to exudation from the tissue surface, otherwise, the tissue would be damaged due to desiccation. Therefore, a plasma-body fluid system will be formed in these cases and most interactions between plasma and biological cells or tissue will involve a moist or aqueous phase (with a typical thickness of a few hundred microns) [212]. Thus, a better understanding of the biological interactions between gas phase plasmas and liquids is necessary. Also, since in many cases the active plasma species (neutral reactive species, electrons, ions and photons) first interact with biomolecules through the liquid layer, it is essential to perform fundamental studies to understand how plasma interacts with biomolecules including amino acids, DNA and proteins which are the building blocks of cells and organs.

There are two main types of plasma treatments in biomedicine researches: (1) Direct and (2) indirect treatment. In the first type, a biological target is directly exposed to a plasma source and the plasma-generated species directly interact with the target. In this case, the living tissue or target often functions as one of the plasma electrodes. This type of direct treatment usually permits a flux of various uncharged species as well as UV radiation to the surface of the biological target. Moreover, an important feature of direct plasma treatment is the existence of a significant flux of negatively and positively charged species reaching the surface of the sample. The efficacy of this method is in close relation to short-living species such as singlet oxygen ^1^O_2_ and radicals such as O_2_^•−^, OH^•^, and atomic radicals which are directly formed by the plasma [207]. The second treatment type involves the pre-treatment of aqueous media, which are in a second step applied to cells or tissue [207]. In this case, the effects involve changes in the solution’s pH and conductivity which affect the reactivity of chemical species, or post-plasma reactions whereby degradation continues for a long time after the plasma treatment. For this method, the formation of relatively long-living species such as H_2_O_2_ and O_3_ as well as secondary radicals generated in the liquid phase is the most essential phenomenon. For both types of plasma treatments, there are however still many challenging issues including (1) a detailed characterization of the plasma chemistry and its effect on the exposed organic materials; (2) a thorough understanding of the plasma generation in and in contact with liquids; (3) an improvement of the design of plasma sources intended for biomedical applications; (4) finding accurate modeling for plasma and living matter interactions; (5) resolving the mechanisms of plasma and living matter interactions, etc. [213]. To move ahead in the further development of actual commercial tools that will be used in hospitals and in finding novel and perhaps even unexpected uses of these plasmas, a better understanding of the mechanisms of interaction of non-thermal plasmas with living organisms, tissues, and cells becomes essential. Obviously, these mechanisms are very complex, owing partly to the complexity of the plasma phase, but mainly to the complexity of the biological targets. Moreover, further complexity is also added due to the presence of liquids surrounding the cells/tissue.

A very promising use of non-thermal plasmas in medical science is wound healing and blood coagulation. Wound repair processes typically involve various intracellular and intercellular pathways activating cellular components of the immune system, the blood coagulation cascade, and the inflammatory pathways. Many types of cells undergo marked changes in gene expression and phenotype, leading to cell proliferation, differentiation, and migration [214]. The blood coagulation process typically involves platelet activation and the coagulation cascade, which can effectively stop bleeding [215,216,217,218]. One frequently used plasma device to control bleeding in endoscope’s surgery is a thermal Ar plasma coagulator [219,220], however, this device is not suitable for external applications because of its small treatment area and relatively high operating temperature. Indeed, for external applications such as wound treatment, it is desirable to operate with non-thermal plasmas which can cover a large treatment area without damaging the surrounding tissue.

**Figure 12:**
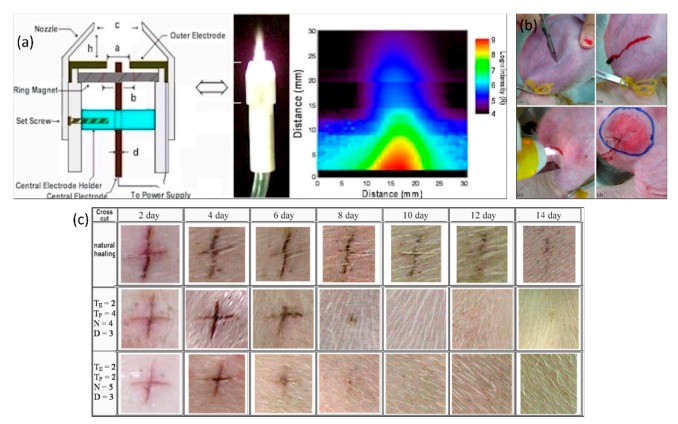
(**a**) The portable plasma spray designed by Kuo [218], (**b**) cutting an artery and leaving the bleeding to stop normally (top row); with plasma treatment the bleeding stops in half time (lower row) and (**c**) comparing the recovery progress of untreated (row 1) and plasma-treated (rows 2 and 3) cross cuts. Reprinted from [218].

In 2012, Kuo [218] designed a portable plasma spray for bleeding control and wound healing which is presented in Figure 12a. These researchers directly treated blood samples using an AC air plasma spray and their in vitro assays verified that the observed plasma blood clotting was not caused by thermal effects (the measured air plasma effluent temperature was less than 55 °C (328 K)). Moreover, a magnetic field also rotated the discharge preventing the formation of arcs and hot spots on the electrode surfaces. It was claimed that the blood clotting is associated with a reactive atomic oxygen flux in the plasma. They observed that with a rapid blood clotting by plasma effluent, the platelet count decreases. Consequently, they concluded that platelets were fragmented by oxidants produced by the reactive atomic oxygen flux to induce blood clotting and provided a surface for the subsequent steps of coagulation, leading to clot formation. The in vivo results revealed that the plasma spray could rapidly stop external wound bleeding (Figure 12b), which was according to the authors due to oxidants such as OH^•^ radicals and H_2_O_2_, which can affect several key steps of platelet function to enhance platelet aggregation, leading to blood clotting. Post-plasma treatment observation of wound healing (Figure 12c) also indicated that the plasma effluent had a positive impact on wound healing and that there were no apparent side effects on the irritated skin. They concluded that most likely the reactive atomic oxygen in the plasma effluent reduces the demand of oxygen used in respiratory burst and indirectly increases the oxygen content of the tissue. Plasma treatment can raise the tissue oxygen tension in the wound site during treatment and thus increases the oxygen supply from the neighboring tissue for wound healing and cell metabolisms.

In another study on plasma-assisted wound healing, Arjunan et al. [221] used a DBD plasma in ambient humid air which could induce angiogenesis via endothelial cell fibroblast growth factor 2 (FGF-2) release through sub-lethal cell membrane damage. PBS, serum-free medium (SFM), and endothelial cells were treated by the DBD plasma and the authors effectively studied how the reactive plasma species affect the angiogenic response. They observed that the initial high H_2_O_2_/OH^•^ dose is responsible for disinfection of the wound surface, while the later low dose helps cell proliferation and angiogenesis. The authors offered their results as a guide for the development of new plasma devices for angiogenesis in wound healing. The most challenging part of non-thermal plasma application for wound healing is the development of new and more suitable plasma devices. Specific RONS, particularly ROS, in controlled doses, need to be achieved by controlling plasma discharge properties such as electron density, temperature, and gas composition [221]. Additionally, the penetration depth of the plasma-produced RONS using different plasma configurations need to be also more precisely determined.

**Figure 13:**
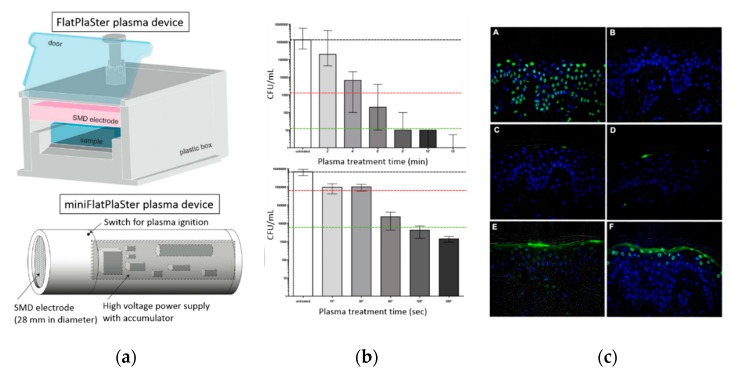
(**a**) Two plasma devices used by Maisch et al. for decolonization of microorganisms on large skin areas, (**b**) decolonization of S. aureus using the two devices and (**c**) histological evaluation of ex vivo porcine skin after plasma treatment: (**A**) positive control, (**B**) negative control, (**C**) immediately after 15 min treatment, (**D**) 24 h after 15 min treatment, (**E**) 48 h after 15 min treatment and (**F**) 48 h of an untreated skin sample. Reprinted from [223].

Besides wound healing, plasmas can also be used for other dermatological purposes. Being a contact-free, painless, self-sterilizing and non-invasive technique, plasma is considered to be a very promising technique in dermatology. The action modes of non-thermal atmospheric pressure plasmas, i.e., bacteria killing, tissue stimulation, anti-inflammatory and anti-itch properties, appear ideal for dermatological applications not only for wound healing purposes but also for skin regeneration and the treatment of diverse inflammatory skin and infectious diseases. The disinfective use of plasma in dermatology is of great interest since non-thermal plasma enables a direct treatment of skin pathogens [222]. In an ex vivo study on a porcine skin model, plasma displayed an efficient lethal effect on wound related bacteria (Figure 13). Plasma treatment caused a decolonization of the tested bacteria without harming cells of the pig skin sample. For this particular study, Maisch et al. [223] applied two different atmospheric pressure plasma devices, FlatPlaSter and miniFlatPlaSter, in ambient air (Figure 13).

Plasma also showed an acceptable performance in skin regeneration in the field of cosmetic medicine. A treatment with a commercial RF N_2_ plasma jet (Portrait^®^ PSR) led to a controlled damage of the skin [224,225,226] and a complete regeneration of epidermis was reported after 10 days. Moreover, an ongoing collagen production, reduction of elastosis and progressive skin rejuvenation was confirmed over one year after the plasma treatment. The patients reported a 60% improvement of their skin texture including wrinkle reduction and skin tone improvements. Plasma treatment was also employed to treat atopic eczema, a very widespread skin disease. The symptoms include redness, swelling, itching, and dryness of the skin. The conventional treatment method is using a moisturizing crème followed by topical anti-inflammatory and anti-microbial treatments [222]. In 2009, Mertens et al. [227] presented a case study on treating atopic eczema with a DBD device using a power of 0.2 W. Plasma treatment was daily applied to the skin for 1 min over a period of 30 days. After this treatment period, the involved researchers observed a reduction of some symptoms such as swelling, itching, and redness. Additionally, no side effects during or after the plasma treatments were reported. For more information on the use of non-thermal plasmas in dermatology, the reader is referred to two interesting review papers: (1) The review paper of Tiede et al. [222] containing a detailed risk analysis for the assessment of two different plasma sources, direct and indirect, in dermatology, and (2) the review written by Heinlin et al. [228] focusing on potential plasma applications in medicine and recent research on skin diseases.

**Figure 14:**
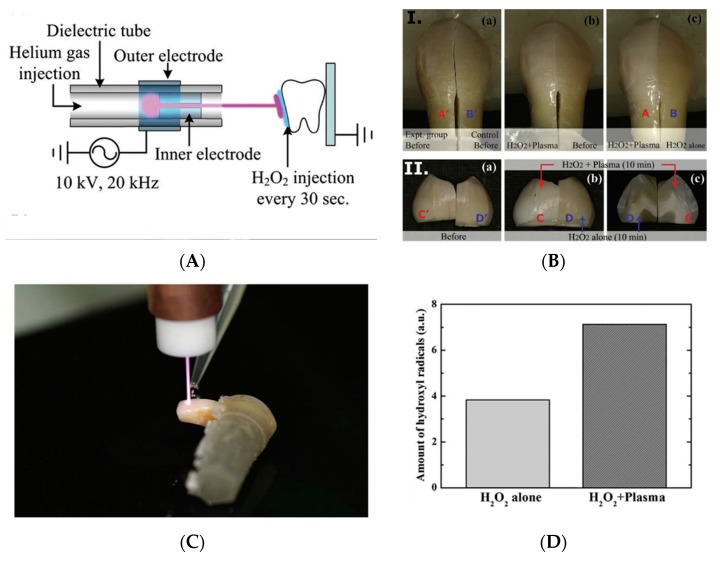
(**A**) Schematic of the plasma device and the tooth bleaching process performed by Lee et al. [234], (**B**) the external bleaching effect of plasma treatment, (**C**) configuration of the tooth bleaching experiment and (**D**) concentration of hydroxyl radicals in the solutions containing tooth before and after plasma treatment. Reprinted with permission from [234].

Besides dermatology, dentistry is also one of the important medical areas in which non-thermal plasma technology has been applied for. The mouth or oral cavity has a characteristic bacterial composition, containing over 700 bacterial species [229]. Dental plaque is a complex oral biofilm made up of hundreds of microbial species organized in communities embedded in a matrix of polymers of bacteria and salivary origin [230]. Some physical and chemical parameters influence the growth and survival of the bacteria in plaque e.g., nutrients, pH, oxygen and redox potential. Therefore, the main concern in plasma dentistry is to investigate the antimicrobial effects produced by plasma to remove dental biofilms and eradicate oral pathogens. It has been shown that reactive oxidative species, charged particles, and UV photons play the main role in the plasma-assisted removal of dental biofilms [231]. Besides biofilm removal, non-thermal atmospheric plasma has also found an important role in tooth whitening and composite restoration. Currently, hydrogen peroxide treatment is used for tooth whitening [232] and it has been stated that hydroxyl radicals generated from the hydrogen peroxide play a main role in the tooth bleaching process [233]. Lately, some researchers have also investigated other possible dental treatments for tooth bleaching and found non-thermal plasma to be a promising candidate. An atmospheric pressure He plasma jet was applied by Lee et al. [234] on some extracted teeth cut longitudinally for tooth bleaching purposes (see Figure 14). Half of the teeth were treated with H_2_O_2_ while the other half of the teeth received plasma and H_2_O_2_ treatments simultaneously for a period of 10 min. The second group was found to show a 3-fold improvement in tooth bleaching compared to the first group (Figure 14). The authors hypothesized that the enhanced bleaching effect may be due to the removal of the tooth surface protein and the higher concentration of hydroxyl radicals induced by the simultaneous plasma treatment (Figure 14). Other research groups also reported similar results: A simultaneous plasma and H_2_O_2_ treatment enhances tooth whitening compared to hydrogen peroxide treatment alone [235]. Another study on the effect of plasma treatment on tooth whitening was carried out using an atmospheric pressure DC micro-jet plasma [236]. The results showed that also by using this plasma source the plasma treatment significantly improved tooth whitening. The involved research group hypothesized that the tooth whitening can be due to the plasma-induced reactive chemical species, formed at the plasma-liquid-tooth interface. In another study, an RF plasma jet was placed in deionized water by Kim et al. [237] and the targeted tooth was also immersed into the water. After an 8 min plasma treatment, color changes were observed on the surface of the treated tooth, which were, according to the authors, mainly caused by the impact of hydroxyl radicals. Similar results on tooth bleaching have also been obtained by other research groups using different plasma devices [238,239,240]. Additionally, there are also some very interesting reviews, particularly the reviews by Hoffmann et al. [231] on the production and application of non-thermal atmospheric pressure plasmas in dentistry and by Arora et al. [241] on dental applications of plasma, where more information on this topic can be found.

### 3.6. Plasma Oncology and Cancer Therapy

A recently emerging and rapidly growing application of non-thermal plasmas in the biomedical field is plasma-assisted cancer therapy. In the past, several studies, which were inspired by plasma-assisted cancer therapy, have already been published [242,243,244]. Many groups proposed an initial model for the mechanisms of interaction of non-thermal plasmas with living cells and tissues in plasma cancer treatment, of which an overview is presented in Table 7. In cancer treatment, two approaches can be considered: Apoptosis or a programmed cell self-death pathway which is an essential issue because cancer cells have frequently acquired the ability to block the apoptosis process and are subsequently more resistant to chemotherapeutic drugs. Contrary to apoptosis, necrosis is an unprogrammed cell death triggered by external factors (or disease). This approach is not an active cell process and does not require energy. Theoretically, the necrosis process can better fend off the development of resistance due to its vigorous nature, however, its cascade can cause inflammation [245].

To investigate the influence of plasma parameters on the detachment or damage of living mammalian cells, Kieft et al. [243] applied an RF plasma needle with He as working gas. Their aim was to minimize the incidence necrosis while efficiently achieve cell detachment which is a sub-lethal cell reaction. They showed that the amount of liquids that covered the cell samples had a critical impact on detachment and necrosis rates, since it directly affects the penetration depth of the reactive species through the liquid. Below a treatment time of 1 min, the cells did not show any time dependence behavior, however, at longer exposure times, cell detachment proceeded linearly until eventually all cells detached. With increasing power, no cell detachment was observed, but increasing power was found to promoted cell necrosis. The authors concluded that UV radiation alone in high doses can cause cell death but cannot induce cell detachment. Additionally, they also neglected the influence of temperature and the applied electromagnetic field. Consequently, the plasma electrons, ions and radicals were discussed as the “suspected” plasma species causing cell detachment or damage. The authors also mentioned that the penetration depth of electrons and ions in the liquid was not sufficient to cause their observed results directly, however, energetic ions in contact with the culture medium may be able to create reactive radicals from water molecules. In another study, Stoffels et al. [244] used the same device, but they treated cells through a gas-permeable membrane to simulate indirect intravenous treatment with a gas plasma-filled catheter. They observed a dose-dependent apoptosis and necrosis in cell lines which was also detectable 6–10 h after plasma exposure. The authors also observed that at low plasma doses (low power and short treatment times) the cells displayed typical patterns for apoptosis such as morphological changes and nuclear DNA condensation. Apoptosis was also reported at higher doses, but in these conditions, necrosis was prevalent. However, necrosis was not instantaneous and thus they concluded that a delayed cell death occurred at higher doses. No immediate cell detachment was reported during this indirect plasma treatment, however, in the case of direct plasma treatment cell detachment occurred even when using very short irradiation times (1–2 s). This means that agents responsible for cell detachment could be blocked by the used membrane.

Inactivation or killing of human Melanoma skin cancer cell lines was demonstrated in an in vitro study by Fridman et al. [242]. They showed that very low doses of floating electrode DBD (FE-DBD) plasma treatment (5 s at 0.8 W/cm^2^) could initiate apoptosis in the cell lines under study. In contrast, higher treatment doses (15 s at over at 1.4 W/cm^2^) caused immediate necrosis and destruction. They also detected the initiation of a complex cascade of biochemical processes, leading to cell death many hours and even days after plasma treatment. After a 5 s plasma treatment, the cells were not immediately inactivated, but the cell growth rate significantly slowed down and the number of dead cells increased 24 h after treatment. It was claimed that the plasma treatment initiated this behavior in cells not through poisoning of the growth media in which the cells reside or through interaction with the aluminum dishes the cells reside in, but through direct interactions between plasma species and cells. However, it was not studied which species generated by the plasma were able to invoke apoptotic biochemical pathways and how these mechanisms were invoked.

The effect of ROS on the apoptosis of human bladder cancer cells using a DC He/O_2_ atmospheric plasma jet was studied Joh et al. [246]. Using a staining assay and flow cytometry, they detected apoptosis changes in the cells after plasma treatment. They observed that both plasma dose as well as the levels of intracellular and extracellular ROS correlated well with the gathered apoptosis rates. The spindle shape of living cells exposed to the plasma was changed to a round shape and detachment of the cells from the extracellular matrix was observed. They mentioned that this detachment might be attributed to plasma-induced damage of cell adhesion molecules. Additionally, the detachment rate was also found to increase with increasing treatment time and applied voltage. Finally, the authors suggested that plasma treatment induces genetic signaling cascade from DNA damage and simple non-genetic physical damage in the cellular membranes. In a related study, Kim et al. [247] used a cold surface-type DBD discharge in ambient air to directly treat mouse melanoma cancer cells. Like Joh et al. [246], these researchers also proposed ROS as key constituents inducing apoptosis. Additionally, the high concentration of ozone generated by the used DBD (~1000 ppm) could also be responsible for DNA damage, according to the authors.

**Figure 15:**
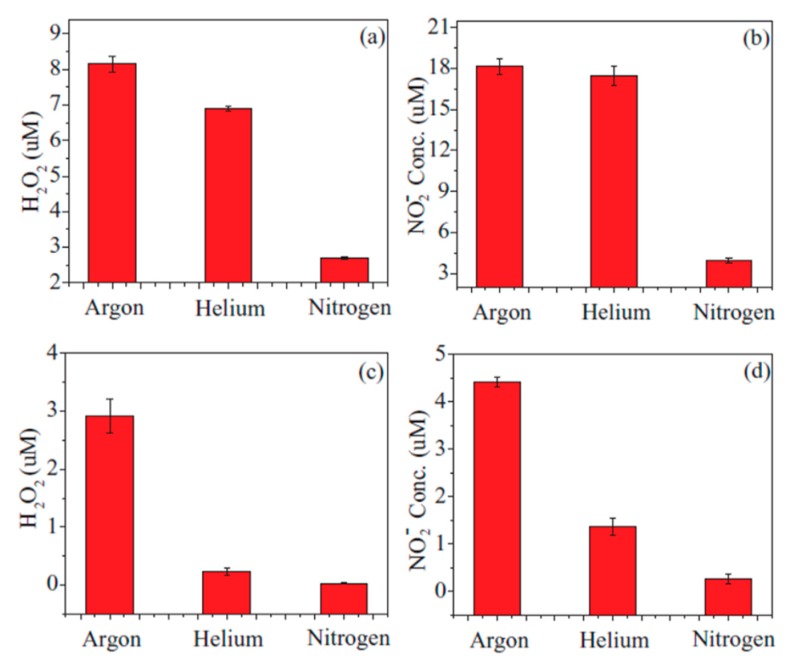
H_2_O_2_ and NO_2_^−^ concentration in plasma discharge generated in deionized water by Keidar et al. [249]; (**a**,**b**) correspond to plasma submerged in deionized water, while (**c**,**d**) correspond to plasma generated outside the water (water volume is 200 mL). Reprinted with permission from [249].

Indirect cancer therapy by using plasma-activated liquids (e.g., water or cell culture media) has also been reported to have some effects on the anti-proliferative activity of cancer cells [248]. An indirect plasma treatment on breast cancer cells was performed by Keidar et al. [249] using a cold plasma jet sustained in Ar, He and N_2_. These authors also suggested that ROS are responsible for the cell apoptosis because measurements indicated that there was a significant difference in H_2_O_2_ concentrations produced by the Ar and He plasma. On the other hand, the concentration of NO_2_^−^ only slightly changed in these two cases. Therefore, ROS (particularly H_2_O_2_) were introduced as an important factor in the interactions of plasma stimulated media with cells (Figure 15). Dobrynin et al. [250] examined both direct and indirect plasma treatments on various cell types including bacterial cells, human cadaver tissue, and mammalian cells (epithelial cells, fibroblasts and melanoma skin cancer cell lines, macrophages, b-lymphocytes) using a floating electrode DBD air plasma. They proposed a hypothesis based on their findings that positive and negative ions have relatively the same effects on biological mechanisms through initiation and catalysis of peroxidation processes. These peroxidation processes are catalyzed by plasma-produced ions both inside and outside of the biological targets, thus they are able to have a greater effect than neutral active species. They mentioned that ROS produced by the plasma are the key components in plasma-cell interactions and that the cell membrane is the first target where the most processes occur on. The thickness of the liquid was also found to be important since the chemical effect resulting from plasma-membrane interactions is dependent on the amount of used liquid. The dosage of plasma treatment was also introduced as one of the main factors influencing plasma-cell interactions since it is closely related to plasma and can be controlled using appropriate plasma parameters. On the other hand, since some biological responses may also result from the plasma treatment, this research group stated that an appropriate knowledge on intracellular biochemistry would be necessary to interpret these responses. The authors also stated that the metabolism of ROS is different in bacteria and mammalian cells. For example, while human cells show protection against species like O_2_^−^, bacteria either lack it completely or their resistance is lower. It was consequently concluded that higher order organisms may have developed more resistance mechanisms to external stress (osmotic pressure changes, ROS, chemical and biological poisons). This research group also observed that plasma treatments selectively interact with some blood constituents to promote blood coagulation through initiation or catalysis of biochemical processes pre-existing in blood. They also observed that plasma treatment can control ion channel activity in cells without destroying or damaging those cells.

Kalghatgi et al. [251] treated mammalian breast epithelial cells using an AC pulsed air DBD plasma and reported that non-thermal plasma created by DBD had dose-dependent effects that range from increasing cell proliferation to inducing apoptosis. As one possible mechanism underlying the observed dose-dependent effects, they proposed intracellular ROS, because at low levels an increased cell proliferation was observed and at high levels cell death was induced through DNA damage. They also studied the effect of medium separately treated with DBD plasma, which was subsequently added to cells, and concluded that there were not significantly different results than in case of direct treatment of cells overlaid with medium. The authors suggested that plasma-induced DNA damage is likely initiated by neutral species and not by charged species produced by the plasma in the gas phase. Additionally, UV was hypothesized to not play a role in plasma-induced DNA damage. Other studies have also shown that the effects of neutral active species cannot be ignored in plasma-cell interactions because neutrals are able to sterilize and they are also responsible for some biological effects (like NO in wound healing) [211,252,253].

Generally speaking, the key role of plasma-generated RONS in oxidation-reduction (redox) biochemistries in cancer therapy is well established in the literature. Moreover, there are some very interesting, comprehensive reviews on this topic, e.g., the review by Schlegel et al. [254], summarizing the progress in applying non-thermal plasmas to tumors, and the review written by David Graves [255] exploring the possible mechanisms and opportunities of the plasmas as a novel anti-cancer therapy. For more information on plasma-assisted cancer therapy, the reader is referred to these review papers.

### 3.7. Plasma Agriculture and Food Safety

Recently, PLIs have also been employed in agricultural applications, with a main focus on seed germination. The effects of UVA and UVB radiations on plants have already been intensively studied [265,266,267,268], however, there are to date only a limited number of studies available in literature dealing with agricultural applications of non-thermal plasma technology. In these studies, non-thermal plasma technology has been leveraged for advantageous physical and/or chemical treatment of crops, seeds and soil [269]. The reactive nature of plasma makes it a suitable technology for seed germination, seed disinfection, plant growth, insect control, retention of quality of agricultural products, etc. [269]. Applications in this context can be classified into pre-harvest and post-harvest practices. Whereas post-harvest utilization of plasma is discussed as food safety, plasma agriculture deals with pre-harvest administration including the remediation of soil, plasma treatment of crops and seeds for decontamination purposes or germination and growth enhancement and the production of plasma-activated medium as nitrogen-based fertilizer. For a more elaborated discussion on these topics, the reader is referred to [269,270,271]. Soil remediation aims for the decomposition of contaminants such as pesticides, various types of industrial waste and bacterial contamination, in order to improve the soil quality. Since soil is characterized by a mutable moisture content, many of the insights from Section 3.3 and Section 3.4 are also relevant in this context.

Although the first experiments of plasma in contact with seeds were performed by Lichtenberg and go back to the eighteenth century [272], the scientific community had to wait until the end of the twentieth century for the first reports on seed treatment with plasma for agricultural applications. In 1994, Krapivina et al. [273] received a US patent for the enhanced germination and crop yield of soybean by means of a glow discharge. They found that the seed surfaces became more hydrophilic after plasma treatment, increasing the water uptake with more than a factor 3 and enlarging the amount of germinating seeds. Six years later, Volin et al. [274] used, on the other hand, a rotating plasma reactor with two different hydrophobic source gases, carbon tetrafluoride and octadecafluorodecalin, to coat seeds with hydrophobic macromolecules for a delayed germination. Additionally, Lynikiene et al. [275] studied the influence of a corona discharge on various seed germination dynamics. They chose low viability seeds of carrot, radish, beetroot, beet, and barley for their study and reported that a corona discharge speeds up seed germination and increases the germination compatibility. RF non-thermal air plasma was applied by Bormashenko et al. [276] to treat beans (Phaseolus vulgaris) and to investigate its impact on the wetting properties and water imbibition of the beans. It was observed that the water absorption rate of plasma-treated beans markedly accelerated. This group also reported that the final percentage of germination was almost the same for plasma-treated, untreated and vacuum-pumped beans. However, the speed of germination was noticeably higher for the plasma-treated beans.

**Figure 16:**
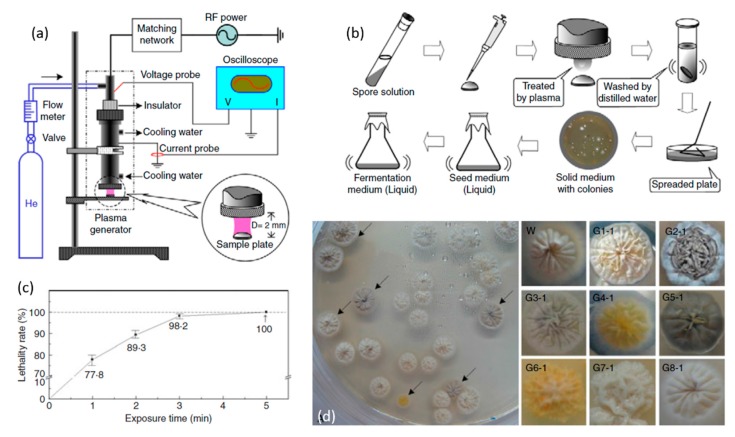
(**a**) The atmospheric radiofrequency (RF) atmospheric pressure He glow discharge used by Wang et al. [277], (**b**) schematic presentation of their mutation breeding protocol, (**c**) variation of the lethality rate with plasma exposure time and (**d**) images of the colonies before and after mutation; (left) a cultivated plate of the strains from plasma-treated spores, (W) a colony from the untreated wild strain, (Gm-1 (where m = 1–8)) the selected mutants from plasma-treated spores. Reprinted with permission from [277].

Plasma technology can also be used in agriculture by mutation breeding methods as described in [277,278]. An atmospheric pressure He glow discharge plasma jet was employed by Wang et al. [277] to generate mutations in the spores of Streptomyces avermitilis which are major antiparasitic agents used commercially in agriculture (see Figure 16). They reported 21% positive mutation rates on S. avermitilis and high productivity and genetic stability of the mutated strain. In another study, Padureanu [278] used a gliding arc discharge to treat seeds of Triticum aestivum L. The cells division in the seeds was studied in the absence and presence of water vapor to highlight the role of water vapor in the non-thermal plasma upon the cells mitotic. It was found that the plasma without water vapor had an inhibitory effect on mitotic division in root meristems of wheat seeds, reducing the value of mitotic index. Also, it was mentioned that chromosomal aberrations induced by the plasma in the absence of water vapors were insignificant in frequency. These observations were explained by the absence of water vapor during the plasma treatment and it was stated that the presence of water vapor during plasma treatment has a major role on cell mitogen which potentiates the mutagenic effect of plasma treatment.

Soil remediation using plasma technology was also examined in some research papers [269,279,280,281]. In a study by Wang et al. [282], p-nitrophenol pollution was decomposed faster in moist soil as compared to dry soil when using a DC air pulsed discharge. This difference was attributed to additional H_2_O_2_ and OH^•^ radical formation in the presence of water. More specifically, Lou et al. [283] found an optimal moisture content of 10% for the degradation efficiency of chloramphenicol-contaminated soil with a DBD plasma source. Soil treatment with this DBD also resulted in a lower bacterial content, inhibiting possible plant diseases [284]. While mineral content did not remarkably change after treatment, the nitrogen content of the soil was strongly increased.

**Figure 17:**
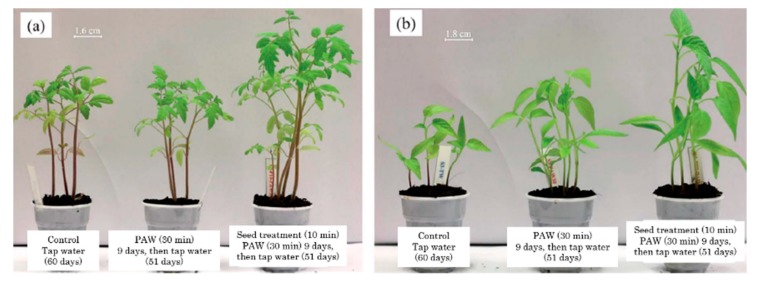
Long term effect of seed treatment by plasma and PAW on (**a**) tomato and (**b**) pepper plant growth. Reprinted from [286].

It has also been illustrated by some research groups that PAW can be a noteworthy candidate as nitrogen-based fertilizer due to its considerable intervention effects against food-borne pathogens [285,286]. Effect of water treated with different atmospheric plasmas on germination, growth rates and overall nutritional value of various plants was studied by Park et al. [285]. They employed three different plasmas: a thermal spark discharge, a non-thermal gliding arc discharge and a transferred arc discharge to show that the used plasma type strongly affects the chemical composition of the plasma-exposed water. The germination rate and plant growth of radish, tomato, and sweet pepper was investigated by Sivachandiran et al. [286] using air DBD plasma-treated water. The combined effect of plasma-treated seeds and PAW was also studied by this group. They concluded that the NO_3_^−^ species produced by the plasma in the water were mainly involved in the cell metabolic process and that the acidic water obtained after plasma treatment could be used as a fertilizer. The seeds germination efficiency and the concentration of NO_3_^−^ and H_2_O_2_ produced in the water was also examined as a function of plasma treatment time. Although a positive effect was observed in terms of germination and seeding growth when combining plasma-treated dry seeds and PAW (see Figure 17), the effect became negative when using plasma-treated wet seeds. Zhou et al. [287] used atmospheric N_2_, He, air and O_2_ micro-plasma arrays to assess the effects of plasma treatment on seed germination and seedling growth of mung bean in aqueous solution. The feed gas and treatment time were found to be important process parameters. Air plasma was the most efficient plasma in improving both the seed germination rate and seeding growth, which was attributed to solution acidification and seed interactions with RONS generated by the plasma.

Besides the above-mentioned pre-harvest utilizations of plasma, plasma technology is also being explored as a post-harvest practice. Currently, developing methods for improving the efficiency and sustainability of the food supply chain and for reducing food waste and energy consumption are major incentives within the field of food science and technology. More specifically, there is at this moment an increasing demand for high quality foodstuffs which are free from undesirable microorganisms and which have a more extended shelf-life. Additionally, these high quality foodstuffs can also reduce food-related illness, which still is a highly significant problem [288]. For instance, in many European countries, foodborne illness caused by Listeria monocytogenes is even an increasing issue [289]. To improve the level of food safety, non-thermal atmospheric pressure plasma processes have proposed to be a prospective alternative to traditional food processes such as heat pasteurization and sterilization which suffer from undesired alterations of food quality [272]. Some other non-thermal technologies including chemical treatment, ozonation, UV irradiation, high pressure processing, and ultrasound treatment have also been explored [290,291]. However, some disadvantages of these latter techniques such as high application costs, requirement for specialized equipment, extended processing time, the generation of undesirable residues, and low efficiencies have made plasma treatment an emerging non-thermal technology for food industry [292].

In a general study, Marsili et al. [288] reported on the use of a high voltage pulsed plasma process to inactivated food-related microorganisms in liquids. Several Gram positive (G+) and Gram negative (G−) microorganisms including *E. coli* (G−), *S. aureus* (G+), *S. enteritidis* (G−) and *B. cereus* (G+) were investigated in this study. The authors concluded that it was difficult to predict whether Gram positive bacteria would be more susceptible to the plasma process than Gram negative types. Their results showed that *S. enteritidis* (G−) and *S. aureus* (G+) were similarly affected by the plasma discharge, while Gram positive *B. cereus* was found to be the most susceptible microorganism. Reasons for this behavior were, however, not presented as it would require further investigation. Recently, Yasui et al. [293] also investigated if plasma was capable to inactivate microconidia of *F. oxysporum* which causes Fusarium wilt of spinach. They examined sterile distilled water and a nutrient solution as solvents. The decontamination capability of different types of gases (air, O_2_, N_2_, He, and Ar) was studied and it was found that O_2_ plasma had the highest decontamination capability, however, ozone generation, which inhibits plant growth, was a negative side effect in this case. On the other hand, air and N_2_ plasmas provided low decontamination performance. Inert gases also showed appropriate decontamination capability in combination with a low production of ozone. They concluded that the presence of O^•^ and OH^•^ radicals in the solution may significantly contribute to the decontamination process. Besides the above-mentioned more fundamental studies, plasma treatments have also been performed on numerous real food products, of which an overview can be found in Table 8.

As shown in this table, inactivation of microorganisms in liquid foodstuffs (mainly milk and juice) using plasma-based processes has been extensively investigated in the past few years with excellent inactivation results; however, at this moment, there is still important information lacking on the impact of plasma on food-related quality attributes. However, knowledge on quality variations is highly essential as the complete liquid may be subjected to possible degradation reactions since the penetration depth of plasma-activated species is typically significantly higher in liquids compared to solid surfaces [272]. Luckily, some authors have already examined the impact of plasma treatment on a few chemical and physical properties of liquid foodstuffs. For example, Gurol et al. [294] used an atmospheric pressure air plasma for testing its ability to decontaminate *E. coli* in milk (commercial ultrahigh temperature (UHT) and raw milk). Their results showed a significant decrease in the number of colonies in milk by more than a threefold log reduction without significantly affecting pH and color of the milk. However, a slight change in milk color occurred after a prolonged plasma treatment time of 20 min. Korachi et al. [301] used the same plasma device to investigate the possible biochemical changes to the proteins, free fatty acids, and volatiles profiles of raw milk samples after plasma treatment. Their results showed no significant changes to the lipid composition and the total ketone or alcohol levels of milk. However, plasma treatment did cause a significant increase in the total aldehyde content. Kim et al. [295] also reported on the microbial safety of milk after DBD plasma treatment. After treatment, they detected no viable *E. coli*, L. monocytogenes and S. typhimurium cells in the treated milk samples. They also concluded that DBD plasma treatment for less than 10 min could improve the antimicrobial properties of milk, however, some slight changes were observed in the milk physicochemical properties after treatment.

Most of the studies dealing with plasma-induced effects on liquid food properties mainly focus on the impact plasma has on specific proteins or enzymes [272]. For example, Takai et al. [296] applied an atmospheric pressure He/O_2_ plasma jet to hen eggs white lysozyme, as a model enzyme in aqueous solution. Results showed a decreased enzymatic activity and structural change of lysozyme, affected by the pH value of the solution, after conducting the plasma treatment. The authors also concluded that these effects were caused neither by UV light nor by plasma heat and suggested that mainly the reactive species generated by the plasma jet affect lysozyme. In another study, Tammineedi et al. [297] tested the efficacy of a non-thermal Ar plasma treatment in reducing the allergenicity of major milk proteins, namely α-casein, β-lactoglobulin, and α-lactalbumin. In this work, no significant effects were noticed on the proteins after conducting plasma treatments. Additionally, Pankaj et al. [298] reported on the effect of an atmospheric air DBD plasma on the inactivation of tomato peroxides, as a model enzyme, in function of treatment voltage and time. It was shown that both voltage and time have a significant effect on the enzyme inactivation. Following a sigmoidal logistic function, peroxidase was completely inactivated after a 5 min plasma treatment.

Besides treating milk and specific proteins or enzymes, considerable efforts have also been done to expose different juices to a non-thermal plasma, as can be seen from the examples given in Table 8. Shi et al. [299] analyzed the resulting Vitamin C content in low temperature air DBD-treated orange juice and also examined the effect of the plasma on microbial strains that were inoculated in freshly squeezed orange juice. They found that the used plasma was effective in inactivating microorganisms which was most probably due to the pH decrease of orange juice induced by the plasma. They also hypothesized that charged particles and RONS species played an important role in the microbial inactivation process. In another work, Kovacevic et al. [302] applied a single-electrode atmospheric Ar plasma jet to treat pomegranate juice. Results confirmed that the plasma had a positive effect on the anthocyanins stability, indicated by an increased anthocyanin content. Additionally, it was observed that the color of the juice varies with Ar flow rate, but not with treatment time nor sample volume. These authors also used the same plasma system to treat chokeberry juice and monitored in this case the changes in polyphenol components after plasma treatment [304]. Ma et al. [303] used a non-thermal bubbling discharge system to treat and sterilize freshly squeezed tomato juice samples. They analyzed the volatile components of treated juice samples by gas chromatography–mass spectroscopy (GC–MS) and observed that some new substances such as anti-2-hexene aldehyde were produced by the plasma treatment while some other substances such as ethyl laurate were destroyed. The authors also claimed that the performed plasma treatment had no significant effect on the smell of tomato juice. Finally, Garofulic et al. [306] used a single-electrode Ar plasma jet to evaluate its effect on the content of anthocyanins and phenolic acids present in sour cherry Marasca juice and compared the results with classical thermal pasteurization and untreated juice. Duration of the plasma treatment and sample volume were reported to be the most important parameters. The authors also showed that compared to both pasteurized and untreated juice, the plasma-treated juice had a significantly higher content of anthocyanins and phenolic acids.

From the paragraphs above, it should become clear that non-thermal plasmas definitely have potential for the treatment of (liquid) foods. At this moment, studies are being undertaken to identify the plasma species which are most lethal to microorganisms, so that, once identified, operating conditions can be selected to get optimal results. One usually unconsidered aspect of plasma-based food treatment is that treatment must be proven not to negatively affect the food organoleptic and nutritional characteristics. Unfortunately, to date, only very limited investigations on this aspect of plasma treatment have been performed, which means more extensive study in this essential field will be required in the near future. Another important challenge this field is confronted with is the scaling-up of the plasma systems. Only by also examining these two unexplored aspects, a breakthrough of plasma technology in food science may be attained.

### 3.8. Plasma in Dielectric Media

Besides the above-mentioned applications, in which non-thermal plasmas are mostly being used in contact with aqueous solutions or solids containing high water amounts, non-thermal plasmas have also been applied in contact with or inside non-aqueous solutions, of which dielectric media are by far the most explored. Indeed, in literature, multiple studies focusing on plasma interactions with non-polar liquids, e.g., transformer oils, mineral oils, hydrocarbon oils, liquefied gases, etc., can be found [307,308,309]. More specifically, electrical discharges in hydrocarbon oils have been widely investigated and electron impact ionization was proposed as the primary mechanism driving a plasma discharge for both negative and positive polarities [310,311,312]. Dumitrescu et al. [312] reported direct ionization for negative pulses (a cathode initiated discharge) as main mechanism, while bubble related ionization was prevalent for anode initiated discharges. The effect of pulse rise time was also investigated by this group as well as by Gournay and Lesaint [313]. The latter researchers studied an anode initiated discharge in cyclohexane and pentane using pulses with rise times varying between 10 and 200 ns. The authors found that the inception of filamentary streamers was preceded by the formation of dark features near the electrode tip. Additionally, the initiation voltage of the streamers was found to be unaffected by the used rise time, while the inception voltage of filamentary streamers was observed to be increasing with increasing rise time.

**Figure 18:**
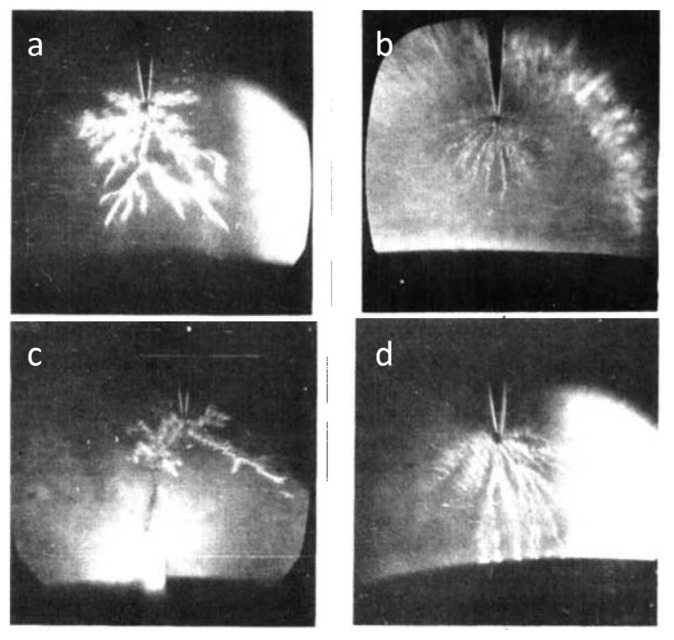
Typical Schlieren images of the pre-breakdown disturbances between point-plane electrodes in pure transformer oil; (**a**) negative point: 60 kV and 2 µs pulse duration, (**b**) positive point: 45 kV and 2 µs pulse duration, (**c**) negative point: 45 kV and 9–6 µs pulse duration and (**d**) positive point: 45 kV and 2–7 µs pulse duration. Reproduced by permission of the Institution of Engineering & Technology [30].

Besides examining the pre-breakdown phenomena, usually called “streamers”, in hydrocarbon oils, these phenomena have also been profoundly examined in mineral transformer oil (see Figure 18) due to the wide usage of this liquid as dielectric medium in high voltage devices [26,30,314,315,316]. More specifically, mineral oil has been used for almost more than a century as insulating and coolant material in transformers due to its excellent thermal and insulating features [317]. As theories on pre-breakdown phenomena in oil were generally restricted to uniform fields, many research groups started to study pre-breakdown processes using non-uniform fields. For this purpose, mainly the point-plane configuration has been used because this configuration permits larger gap distances without using excessively high voltages. In this geometry, streamer formation using positive or negative polarities however shows some differences [318]. In the beginning, the large majority of studies focusing on the pre-breakdown mechanism in mineral oil involved the use of relatively small gap distances. For instance, in 1981, Devins et al. [26] applied a point-plane reactor with gap distances up to 2.5 cm. In one of the early studies carried out by Hizal et al. [319] using a 0.3 cm gap distance, it was shown that positive discharges initiate with a greater time lag, but propagate at a much faster rate compared to the negative discharges under the same conditions. The authors also found that the discharge propagation rate is very sensitive to the magnitude of the applied voltage. Additionally, these researchers observed that in the supersaturated oil they used, the discharge regions grew until they reached the plane electrode, which contradicted the gas-bubble breakdown mechanism in which, after growing to a critical size, a gas discharge within the bubble would lead to total breakdown. The authors suggested that in their case the charge multiplication in the discharge regions and the electric field enhancement in front of the discharge channels were the most important factors in the mechanism, leading to breakdown. From the 1990s on, studies of streamers inside mineral oil using larger gap distances (up to 10 cm) and very high voltages, which are relevant to the conditions in actual transformers, have also been carried out [320,321,322,323,324,325,326]. For example, Saker et al. [327] presented an experimental study of the pre-breakdown mechanism in mineral oil at large gap distances (up to 35 cm) in a point-plane geometry and studied the propagation of positive and negative streamers. Using positive polarity, the pre-breakdown was qualitatively similar to the breakdown observed at shorter gap distances (20 cm). In contrast, using negative polarity, breakdown voltages remained higher than in case of positive polarity for distances up to 35 cm. However, pre-breakdown at negative polarity presented many qualitative similarities with positive polarity, such as a continuously luminous propagating head and periodic re-illuminations. Additionally, in 2001, Massala and Lesaint [328] investigated the propagation of streamers in mineral transformer oil also using large point-plane gaps (up to 35 cm), under impulse voltage. They observed that breakdown voltages were not very different for negative and positive polarities when using large gap distances (larger than 10 cm) and also observed that both polarities showed many similar features. These results were thus very different from the ones obtained by other research groups, which typically observed considerably higher breakdown voltages when using negative polarity compared to positive polarity. The authors also observed that both polarities showed many similar features, but at the same time, also some characteristic differences which mainly resulted from the propagation mechanisms at the streamer head. Positive polarities were found to propagate faster and longer and the propagating streamer heads were also found to emit less light when using positive polarity.

**Figure 19:**
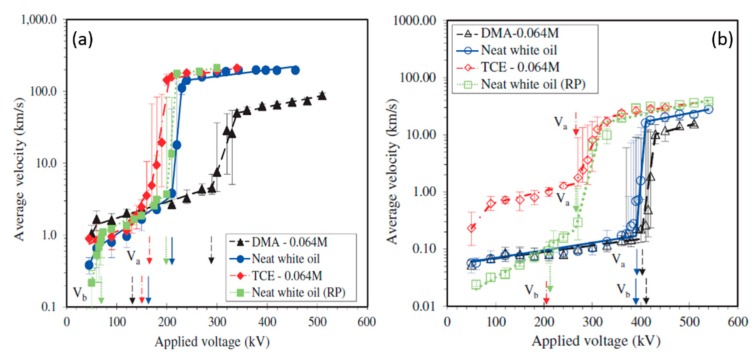
Average velocity of (**a**) positive and (**b**) negative streamers versus applied voltage [RP: reduced pressure]. Reprinted with permission from [334].

Besides plain mineral oils, streamers in highly-refined mineral oils with additives have also been investigated [26,320,321,326,328,329,330]. As previously mentioned, mineral oil has excellent electrical insulation properties because of high acceleration voltages for fast streamers. This high acceleration voltage is possibly related to the presence of poly-aromatic compounds inside mineral oils. However, by refining mineral oil, it is possible to change the content of the electronically active compounds such as aromatics, leading to a potentially different dielectric behavior. Therefore, white oils, which are highly refined and hydrogenated mineral oils virtually free from poly-aromatic compounds, can be considered as appropriate very low aromatic model liquids. Additionally, also blends of white oil and additives can be considered as appropriate models of mineral transformer oil [331,332,333]. Because of the different dielectric behaviors of traditional mineral oils and modern dielectric liquids (such as white oil), Dung et al. [334] performed a worthwhile experimental investigation, using an 8 cm long point-plane gap, to address the underlying reasons for the different dielectric behavior. First of all, it was revealed that reduced pressure significantly accelerated both positive and negative streamers. Secondly, the authors investigated the effect of two additives, *N*,*N*-dimethylaniline (DMA) as a low ionization potential additive and trichloroethylene (TCE) as an electron scavenger, on the behavior of the streamers (Figure 19). DMA strongly influenced positive streamers: It changed streamer velocity and reduced the breakdown voltage but at the same time increased the acceleration voltage where breakdown streamer velocity drastically increased. In contrast, TCE influenced both positive and negative streamers, however, it mainly increased the velocity of negative streamers and resulted in a reduction of both breakdown and acceleration voltages. Based on these observations, Dung et al. concluded that positive streamers were more dangerous than negative ones in white oil. However, the hazard of positive streamers at lightning impulse was significantly reduced for mineral transformer oil. The authors hypothesized that this observation could be caused by the fact that the low ionization potential property of aromatic/poly-aromatic compounds slightly changed the streamer velocity and the breakdown voltage and largely increased the acceleration voltage of positive streamers. For negative streamers, in contrast, the electron trapping property of these (poly-)aromatic compounds increased the velocity by a factor of ten and reduced the breakdown and acceleration voltages by 50% and 30%, respectively. The researchers also observed that positive streamers behaved like those in mineral transformer oil after adding DMA while the same influence was achieved for negative streamers after adding TCE. It was therefore proposed that adding both additives to white oil could produce a type of liquid similar to mineral transformer oil for both polarities. A sufficient difference in ionization potential between aromatic/poly-aromatic compounds and paraffinic/naphthenic compounds was considered to be the main reason for the very high acceleration voltage of mineral transformer oil, thus, aromatic/poly-aromatic compounds are play a crucial role in the electrical insulation properties of mineral transformer oil. In another, closely related study, Lesaint and Jung [331] have investigated the influence of pyrene, an aromatic additive, on the propagation of positive filamentary streamers and breakdown in liquid cyclohexane with a point-plane electrode using gap distances up to 5 cm. These researchers concluded that by the addition of pyrene, the propagation was facilitated and therefore breakdown voltage was lowered. Streamers also became much more branched and a much larger number of filaments propagated than in pure cyclohexane. This observation was correlated to a significant increase of the acceleration voltage of fast streamers at high voltage.

From 2009 on, some studies on vegetable-based oils and biodegradable liquids (e.g., natural ester liquids) have also been carried out to find new liquids which could potentially replace mineral transformer oils in the future [335,336]. Ester liquids are considered to be excellent alternatives as they have a better environmental performance and a higher fire safety guarantee than mineral oils [336]. Tran Duy et al. [335] experimentally studied streamer formation and breakdown phenomena in natural esters by carrying out experiments with gap distances varying from 2 to 20 cm. Several propagation modes were observed, which showed a transition from “slow” (velocity of approximately 1 km/s) to “fast” (velocity up to 200 km/s) streamers. Their results indicated that lower breakdown voltages and shorter time to breakdown are required in ester liquids compared to mineral oils. Liu et al. [336] also performed a similar investigation using both synthetic oil and natural ester liquids with gap distances varying between 1.5 and 10 cm. They also found that streamers in both natural and synthetic esters propagate faster and with more branches than in mineral oil. Additionally, ester liquids also showed lower breakdown voltages than mineral oil because of their low tolerance to fast streamers, which was also in agreement with the results obtained by Tran Duy et al. [335].

Finally, heavy oil has also been modified by plasma in the last decade as a promising conversion method for different kinds of hydrocarbons [337,338,339]. Crude heavy oil has been submitted to a DBD plasma treatment using different gases and various treatment times by Honorato et al. [340], after which the properties of the heavy oils were characterized and the basic sediments and water contents of the oil was determined using ^1^H low and high field nuclear magnetic resonance (NMR). No significant changes in the viscosity of the oil fraction and no changes in the chemical shifts associated with any of the oil components were observed after plasma treatment. The major effect caused by the plasma treatment was the extraction of water with a consequent drop in viscosity of the water-oil emulsion.

A clear overview of different studies focusing on the generation of plasma electric discharges in dielectric media can be found in Table 9.

### 3.9. Plasma in Polymeric Media

Besides the generation of plasma in dielectric media, plasmas have also been recently applied in and in contact with polymeric solutions, which is a relatively new application of non-thermal plasma technology. The main aim of exposing polymer solutions to plasma is to increase their electrospinnability and generate nanofibers with enhanced fiber morphology [345,346,347,348,349,350]. It should be noted that plasma technology has already been widely used to enhance the surface characteristics of electrospun nanofibers as a post-electrospinning treatment method [351,352,353,354,355,356,357]. However, it is also well known that a polymer solution should have appropriate characteristics to be electrospinnable since the morphology of the resultant fibers is directly affected by the polymer solution properties. As such, preparation of an appropriate polymer solution is one of the key challenges in an electrospinning process. Taking this into account, plasma technology has been employed by a few research groups to perform pre-electrospinning plasma treatments (PEPT) as an innovative and environmentally friendly method to change polymer solution properties in an effort to increase the electrospinnability of these solutions [345,346,347,348,349,350].

The first group reporting plasma treatment of a pre-electrospinning polymer solution was Shi et al. [348,349]. They have used an atmospheric pressure capacitively-coupled He DBD to treat polyethylene oxide (PEO) and polyacrylonitrile (PAN) solutions and observed that the polymer solution viscosity, conductivity and surface tension increased after plasma treatment and consequently finer and smoother nanofibers were formed with fewer microbeads and increased crystallinity upon electrospinning. In a first study, these authors provided positive results of the plasma treatment on the electrospinnability of PEO/water solutions. In a subsequent study, the same researchers examined the antibacterial activity of electrospun Ag/PAN nanofibers, prepared from PAN solutions exposed to the same plasma system. *E. coli* and B. cereus bacteria were used in an antibacterial assessment assay and hybrid Ag/PAN nanofibers showed complete inhibition of both microorganisms. The authors also tested the durability of the antibacterial activity of the Ag/PAN nanofibers prepared from plasma-treated solutions and observed slow and long-lasting Ag^+^ ion release.

In another study by Colombo et al. [347], three different plasma sources were used to promote the electrospinnability of poly-L-lactic acid (PLLA) solutions in dichloromethane (DCM). They observed better results with the exposure of the polymer solutions to a single electrode nano-pulsed Ar plasma jet which was generated on top of the solution. In contrast, when direct liquid phase and gas phase discharges were used as plasma sources, a spark regime was observed which caused polymer degradation and subsequently poor electrospinnability of the plasma-treated solutions. These researchers also detected OH^•^ radicals, N_2_* and N_2_^+^ species in the gas phase plasma and assumed that these species together with other charged molecules led to an increase in solution conductivity and consequently improved solution electrospinnability.

**Figure 20:**
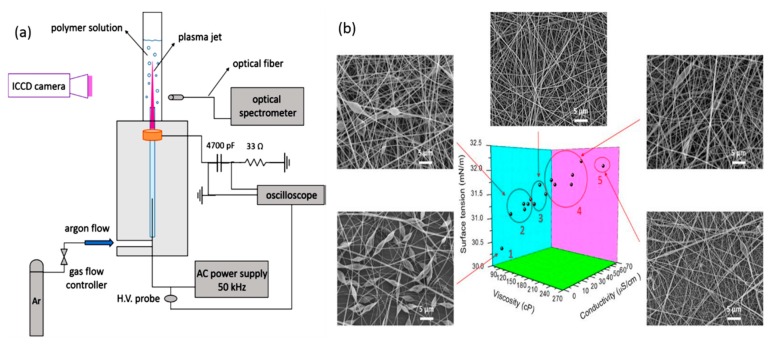
(**a**) Schematic representation of the atmospheric pressure argon plasma jet directly submerged into the liquid phase for PEPT used by Rezaei et al. [345,346], (**b**) 3D image of the physical properties and SEM images of PLA nanofibers produced from plasma-treated solutions. Reprinted from [345].

In the above-mentioned preliminary studies, the discharges were mostly generated above the surface of the polymer solutions. Inspired by the above-mentioned works but using a novel plasma configuration, an atmospheric pressure plasma jet directly submerged into the polymer solutions, researchers started to investigate the plasma treatment of poly-ε-caprolactone (PCL) solutions in a mixture of chloroform (CHL) and *N*,*N*-dimethylformamide (DMF) and also observed a significantly improved PCL nanofiber morphology as a result of PEPT [350]. Grande et al. [358] also studied the potential of PEPT for fabrication of continuous 300-Polyethylene oxide-polyethylene oxide terephthalate/polybutylene terephthalate (PEO PEOT/PBT or Polyactive) nanofibers. These researchers investigated the effect of performed plasma treatments on the final electrospinnability of polyactive solutions in three different sets of solvent mixtures namely CHL/Hexafluoro-2-propanol (HFIP), CHL/DMF and CHL/methanol. It was concluded that CHL/HFIP was the best solvent mixture for polyactive because a plasma treatment time of 3 min in this case was found to give the highest quality nanofibers. MTT assays (abbreviation for the dye compound 3-(4,5-Dimethylthiazol-2-yl)-2,5-diphenyltetrazolium bromide for measuring the functionality of cells) also revealed that a better cell adhesion occurs on the polyactive nanofibers fabricated from the polymer solutions containing CHL/HFIP and CHL/methanol. Rezaei et al. [345] employed the same in-liquid plasma configuration (Figure 20a) to investigate in detail the effects of PEPT on the physical properties of poly lactic acid (PLA) polymer solutions. Using this plasma configuration, more pronounced effects induced by the plasma treatment on the exposed pre-electrospinning polymer solutions were anticipated. The influence of various plasma parameters such as plasma treatment time, argon flow rate, applied voltage as well as the influence of PLA concentration were thoroughly investigated and it was observed that increasing plasma treatment time, gas flow rate and applied voltage considerably influences the physical properties of PLA solutions including viscosity, surface tension and electrical conductivity. Consequently, these plasma-induced physical changes into the solutions led to a significant morphological enhancement of the final PLA nanofibers as shown in Figure 20b. In this study, a time- and space-resolved ICCD imaging was also applied to study the bubble dynamics in the argon plasma-solution system. It was found that the bubble formation during PEPT strongly affects the gas-solution interfaces and as a result affects the charge and energy transfer possibilities between the plasma and the solutions. The PEPT-induced liquid chemistry into the PLA solutions and related organic solvents and its effect on the chemical composition of the resultant nanofibers have also been studied by Rezaei et al. [346] using a broad range of gas, liquid and solid phase analysing techniques. The chemical changes to the solvent molecules and the nature of chemical species which led to the enhanced electrospinning were determined using OES, EPR, NMR, UV–vis absorption and fluorescence excitation-emission matrix (EEM) spectroscopic analysis. They concluded that the main plasma-induced species responsible for the increased conductivity was HCl. Morphological SEM studies of electrospun PLA nanofibers also showed that a complete morphology enhancement only occurs when both polymer and solvent molecules were exposed to PEPT. Their performed XPS measurements also revealed that the surface chemical composition of the electrospun PLA nanofibers was mostly preserved.

Since plasma induces both physical and chemical changes in the polymeric media, investigations of both plasma-induced physical and chemical phenomena are crucial within this innovative research field. Considering the broad range of polymer and solvent types, which can be used for electrospinning, different plasma-induced liquid chemistries are also to be expected, but will require considerably large research efforts in the future to be fully revealed.

## 4. Conclusions and Perspective

In summary, this review paper intended to provide an overview of studies focusing on non-thermal plasma interactions with various liquids ranging from water over transformer oils to biological solutions and liquid food. Most of the applications of the rapidly emerging field of PLIs are based on the generation and interaction pathways of key reactive species in both plasma and liquid phases. At this moment, deeper knowledge of the multiphase processes involved in plasma-liquid systems has already been obtained thanks to the progress in plasma diagnostics and the production of a wide variety of types and configurations of plasma-liquid systems. However, numerous unexplained questions still remain in this exciting research field, which definitely require more profound investigations before they can be answered. In the following sections, some important conclusions and possible perspectives regarding each of the discussed application fields will be given.

It is known, from many contributions already reported, that a wide range of nanomaterials can be successfully synthesized and functionalized using various plasma-liquid systems. However, the reaction pathways and nanomaterial growth mechanisms are not obvious yet and the exchange between plasma and liquid phase requires further research efforts. Larger efforts on basic fundamental studies in this research area will also help with scaling-up and reaching industrially viable nanomaterial processing. Several established instrumental techniques in analytical chemistry also use plasma technology where in some cases the electrical discharge is in contact with the liquid. Next to that, laser-induced breakdown spectroscopy is coming forward as an attractive alternative, also for the analysis of liquid samples. While there is a growing research based on the accuracy of these methods, a fundamental understanding of their principles is still largely lacking. Furthermore, a couple of novel water analyzing methods have been proposed, where the detection of metals occurs from the interpretation of the optical emission spectrum of the electrical discharges in direct contact with the water, opening a new subfield in this area of research.

In general, discharges in water are highly suitable for a wide variety of applications including water purification as well as biological decontamination and generation of shock waves for rock fragmentation and surgery. Despite these successful applications, it was also demonstrated that the erosion of electrodes in pulsed electric discharges in water often results in the production of some metal and oxide NPs. Due to their nanometer sizes, these particles may easily enter the drinking water system, which can in turn present potential danger to the human body. Additionally, because of their small size, it is very difficult to remove these NPs from water. Water purification by means of electrical discharges is one of the most cited applications of PLIs. As an advanced oxidation process, plasma technology has the ability to decompose organic aqueous materials, ideally up to full mineralization, and to oxidize inorganic pollutants. The contribution of various reactive species including the hydroxyl radicals, ozone, atomic oxygen, hydrogen peroxide, hydroperoxyl radicals, superoxide anions, singlet oxygen atoms, atomic hydrogen, and several reactive nitrogen species has been suggested. Unfortunately, studies in literature mostly focus on reaction kinetics and oxidation pathways of organic pollutants whereas the oxidation of inorganic pollutants has been severely less investigated. To bring this technology closer to industrial implementation, more comparative studies will be required which consider the performance of plasma-based approaches in comparison to other advanced oxidation processes or competitive techniques. Moreover, plasma technology for water treatment would also particularly benefit from a detailed toxicity analysis of the effluent water, since many questions on the formation of hazardous by-products such as long-living reactive nitrogen species remain.

Non-thermal plasmas have also been successfully applied for the inactivation of a wide range of bacteria sustained in different liquids. Despite many efforts aimed at understanding and describing plasma-induced sterilization effects in liquid media, it is to this date still not clear which plasma species and in particular which RONS play the most dominant role in bio-decontamination of water as well as in the plasma biomedicine field. Various plasma agents have been identified or predicted to be involved in the decontamination processes in plasma-liquid systems, but there currently still is an unclear image of all involved chemical processes and their correlations with the associated decontamination effects. Although some possible chemical reaction mechanisms were hypothesized by different researchers based on plasma diagnostics as well as gas- and liquid-phase analysis, it is to this date still vague which reaction mechanisms dominantly affect or support plasma-induced acidification and subsequently bacterial inactivation. In general, RNS such as nitric oxide and its derivatives formed with liquid (mainly water) including nitrites, nitrates and peroxynitrites are proposed to be the main reason for the increased acidity of plasma-treated liquids. Additionally, it is also generally accepted that ROS as well as RNS play a dominant role in the sterilization process. However, possible synergistic effects of these processes and post-discharge reactions in plasma-treated liquids due to the presence of longer-living radicals and reactive species, which can be transferred from the gas phase into the liquid phase should also be taken into account. Moreover, it is also essential to find out if the antimicrobial effects are due to the combination of all plasma generated radicals and oxidants in the solution or not. At this moment, the understanding of the interactions between plasma species and bacteria and their action mechanisms is still very limited. Consequently, multiple studies are still required in an effort to obtain advanced fundamental knowledge on this particular application field.

The noticeable potential of plasma applications in medicine has led to the appearance of the term “plasma biomedicine” as a new and independent medical field. Today, research of the effect of non-thermal plasma on tumor cells is being done and some promising results are there, but the simultaneous effect on normal cells has to be studied in depth as well, while validation is also needed for its successful application. At this moment, plasma biomedicine is still in its infancy and more advanced studies are required before plasma technology can be used in a cost effective, efficient and predictable manner in clinical settings. In addition, based on scientific evidences, plasma technology also has a bright future in skin, wound and tooth treatments. However, one of the main challenging features of plasma application in these fields is the development of new and more efficient plasma devices. Additionally, more studies need to be performed regarding the mechanisms of action.

In the field of plasma agriculture, soil reclamation by means of plasma treatment is limited by similar techno-economic challenges as the water treatment applications, namely the high energy demand, the possible formation of toxic by-products and the upscaling of laboratory scale reactors for industrial implementation. In contrast, recently emerging studies in the field of plasma agriculture clearly show that various plasma configurations and plasma types can significantly enhance the growth rate of different seed types. Within this context, plasma treatment parameters such as type of carrier gas, treatment time and input power have to be very accurately determined and optimized for each seed type under study. However, there is still a need for broader studies in this research field focusing on the optimization of plasma treatment parameters and also on the development of accurate seed surface analysis and plant treatment protocols in an industrial setting. Currently available studies also indicate the successful application of non-thermal plasma in food science for the inactivation of pathogens and improvement of safety of food products. However, a few pioneering studies also show some limitations of the plasma treatment such as the acceleration of lipid oxidation and its negative impact on the sensory characteristics of food. More detailed studies are recommended to elucidate the quality aspects of plasma-treated food including sensory characteristics and the retention of these properties during storage in industrial applications. Moreover, comprehensive investigations are still required to investigate the impact of the plasma treatment on the nutritional value of various food products. It is also noteworthy to mention that in some plasma-liquid applications, e.g., plasma agriculture and food safety, there are only partial links with the liquid phase and plasma interactions with the solid phase are also of importance and need to be considered as well.

Besides the treatment of aqueous solutions, non-thermal plasma can also be used in or in contact with dielectric media. Mineral or synthetic oils are mainly being applied in high voltage apparatuses. However, emerging high voltage alternating current and high voltage direct current have prompted researchers to direct their focus onto insulation liquids which can bear the rising high voltage levels. Consequently, new transformers need high quality and high purity insulating oils at the point of use as the increasing voltage typically results in greater electrical stress in insulating materials. To handle these greater stresses, new insulating liquid materials with better dielectric and thermal properties are required, while they should also be environmentally friendly and safe to use. At this moment, vegetable-based oils (white oil and natural ester liquids) are progressively replacing traditional insulating liquids. These safe-to-use, environmentally benign vegetable-based oils are able to provide a higher performance than mineral oil and are known to show definite insulating and thermal gains. Research has already indicated that the oil nature has a strong influence on the electrical discharge shape, its initiation, threshold voltages and propagation velocity. Therefore, intense work on the electrical characterization of discharges inside new dielectric medium candidates is essential. Furthermore, research still needs to be conducted on the characterization of the oil dielectric strength, the aging of the novel molecular liquids, causing degradation of the dielectric strength, the electroosmotic flows in the oils, and the tribo-charging that can lead to electrical failures.

Finally, one of the most recent applications of PLIs is the treatment of polymer solutions prior to electrospinning. Depending on the nature of the solvents and polymers used in preparing the electrospinning solution, optimized plasma treatment conditions should be defined for each particular polymer–solvent combination. Furthermore, plasma configuration and discharge type may be able to considerably affect the final properties of the polymer solution. However, confirmation of this hypothesis will require the use of multiple types of plasma sources for polymer solution treatment, studies which have not yet been conducted at this moment. Additionally, the particular plasma-induced mechanism responsible for the observed enhanced electrospinnability is also not yet revealed. Consequently, this newly emerging application of PLIs is a relatively unexplored research field requiring extensive fundamental investigations in the future to realize a major breakthrough in this particular application field.

In conclusion, the research field of PLIs, with a broad range of promising applications which have been introduced in this review paper, is of growing attention in physics, chemistry, material, and biomedical sciences as well as the food industry. Of course, further progress in this field still needs more sophisticated analytical techniques especially in the liquid phase and at the plasma-liquid interface to be able to more precisely determine the density of plasma-generated chemical species and the chemical pathways. Consequently, based on the obtained knowledge scientists will be able to overcome the present challenges in this field and new applications can be also emerged in the field of PLIs.

## Figures and Tables

**Table 1:** An overview on metal NP synthesis using non-thermal plasma generation in liquid and in contact with liquid.

No	NPs	Size of NPs (nm)	Raw Material	Liquid	Plasma Source	Description	Reference
1	Ag, Au, Ni, Ti	100	Metal wire cathode	K_2_CO_3_	Atmospheric glow discharge	Cathodic plasma electrolysis. Voltage: 120–160 V, time: 10, 30 min.	[71]
2	Ag	3.5	AgNO_3_	Ethanol	Atmospheric pulsed DBD jet	Ar glow discharge on the solution. Voltage: 5 kV, gas flow: 0.3–1 L/min, gap: 3–5 mm, frequency: 67.5 kHz.	[72]
3	Ag	5–50	AgNO_3_	Ultrapure water	Bipolar pulsed discharge	High frequency discharge directly in liquid phase with needle to needle electrode geometry. Voltage: 250 V, pulse frequency: 30 kHz, pulse width: 2 µs, gap: 0.2 mm.	[96]
4	Ag	7–13	AgNO_3_	Water	Atmospheric DC micro-plasma discharge	He plasma on the solution. Voltage: 0–15 kV, gas flow: 25 standard cubic centimeter per minute (sccm), gap: 3–4 mm, time: 30 s–10 min.	[97]
5	Ag	57	Silver wire	Water	Arc discharge	Wire explosion in water. Voltage: 12 kV, capacity: 10 µF.	[74]
6	Al_2_O_3_	10–100	AlCl_3_	Water	Bipolar pulsed discharge	High frequency discharge directly in liquid phase with needle to needle electrode geometry. Voltage: 250 V, pulse frequency: 30 kHz, pulse width: 5 µs, gap: 0.3 mm.	[73]
7	Au	10–180	HAuCl_4_	Water	DC micro-plasma	He or Ar micro-plasma on the solution. Voltage: 0.8–2 kV, current: 1–5 mA, gap: 0.5–1.5 mm, gas flow: 25 sccm, time: 10 min.	[91]
8	Au	20, 50	HAuCl_4_	Water	Pulsed DC discharge	Glow discharge in aqueous solution. Voltage: 1.6, 3.2 kV, pulse frequency: 15 kHz, pulse width: 2 µs, gap: 0.3 mm.	[64]
9	Au	10	HAuCl_4_	Water	Pulsed DC discharge	Ar plasma into the solution. Voltage: 960 V, current: 0.5 A, pulse frequency: 15 kHz, pulse width: 2 µs, time: 10 min, gap: 0.3 mm.	[92]
10	Au	2–10	HAuCl_4_	Water	Pulsed DC discharge	Plasma is generated in the aqueous gold solution with needle to needle electrode geometry. Frequency: 10 kHz, pulse width: 250 ns, time: 30 min, gap: 0.2 mm.	[93]
11	Au	150	HAuCl_4_	Water	Pulsed DC discharge	Discharge directly in liquid phase. Voltage: 1.6, 2.4, 3.2 kV, gap: 0.3 mm, pulse frequency: 15 kHz, pulse width: 2 µs, time: 5–45 min.	[94]
12	Au	32.75, 36.87	HAuCl_4_	Water	DC micro-plasma	He plasma on the liquid. Voltage: 2 kV, current: 8 mA, time: 15 min, gap: 2 mm.	[75]
13	Au	10–65	Gold wire electrode	Water	DC arc discharge	Submerged gold wires in deionized water as electrodes with needle to needle geometry. Voltage: 20–40 V, pulse width: 10 µs, current: 4 A, gap: few microns.	[95]
14	Co_3_C	4–70	Cobalt plate and tip	Ethanol	DC plasma discharge	Ar plasma in the ultrasonic cavitation field. Voltage: 55 V, current: 3 A, gap: 1 mm, power: 165 W, time: 15 min.	[103]
15	Co	10–100	CoCl_2_	Pure water	Bipolar pulsed discharge	High frequency discharge directly in liquid phase with needle to needle electrode geometry. Voltage: 250 V, pulse frequency: 30 kHz, pulse width: 5 µs, gap: 0.3 mm, time: 10–120 min.	[100]
16	Cu	11–26	Copper wire	Ionic liquid	DC discharge	Cathode glow discharge on the liquid in Ar atmosphere. Voltage: 450–500 V, current: 10 mA, time: 30 min, pressure: 100 Pa.	[98]
17	Cu	5–50	CuCl_2_	Ultrapure water	Bipolar pulsed discharge	High frequency discharge directly in liquid phase with needle to needle electrode geometry. Voltage: 250 V, gap: 0.3 mm, pulse frequency: 30 kHz, pulse width: 5 µs.	[99]
18	Fe_3_C, χ-Fe_2.5_C	5–600	Iron tip	Ethanol	Low power DC discharge	Ar plasma in the ultrasonic cavitation field. Voltage: 55 V, current: 1.58 A, gap: 1 mm.	[102]
19	Fe	9.5–21.6	Iron electrodes	Toluene/water	Pulsed AC discharge	Discharge directly in liquid phase. Voltage: 100 V, current: 6 A, gap: 1 mm, pulse width: 10 µs.	[104]
20	Zn	30–200	ZnO powder	Ethanol	Microwave plasma	Plasma in the liquid. Frequency: 2.45 GHz in TE10 mode, power: 235 W, time: 10 min.	[101]
21	Zn	10–200	Zinc plate	Water, alcohol	Microwave plasma	Plasma in the liquid. Power: 250 W, time: 30 s, gap: 1 mm.	[105]
22	ZrC	10	Zirconium electrode	Ethanol	Pulsed AC discharge	Discharge directly in liquid phase. Current: 20 A, pulse frequency: 60 Hz, pulse width: 3 µs, gap: 0–1 mm, time: 1 h.	[66]

**Table 2:** Water solubility and standard oxidation potential of some gas phase species under standard conditions [145,146,147,148].

Chemical	Solubility	Reaction	Oxidation Potential (V)
OH^•^	Half lifetime: few hundreds µs	${\mathrm{OH}}^{•}+H^{+}+e\to H_{2}O$	2.81
O(^3^P)	Lifetime ~10^−5^ s	$O+2H^{+}+2e\to H_{2}O$	2.42
O_3_	2.27 mmol/L or 109 mg/L	$O_{3}+2H^{+}+2e\to O_{2}+H_{2}O$	2.07
H_2_O_2_	Infinite	$H_{2}O_{2}+2H^{+}+2e\to 2H_{2}O$	1.78
HO_2_^•^	Less reactive than the matching anion	${\mathrm{HO}}_{2}^{•}+3H^{+}+3e\to 2H_{2}O$	1.50
O_2_	0.28 mmol/L or 8.9 mg/L	$O_{2}+4H^{+}+4e\to 2H_{2}O$	1.23
O_2_^•−^	–	–	1.00
H_2_O	Infinite	$2H_{2}O+O_{2}+4e\to 4{\mathrm{OH}}^{•}$	0.40
ONOOH/ONO_2_^−^	Acid half lifetime ≈ 20 ms	$\mathrm{ONOOH}+H^{+}+e\to {\mathrm{NO}}_{2}+H_{2}O$	2.10
NO_3_^−^	Fairly soluble	${\mathrm{NO}}_{3}^{-}+4H^{+}+3e\to \mathrm{NO}+2H_{2}O$	0.96
NO	1.55 mmol/L at 37 °C	–	0.90
N_2_	0.49 mmol/L or 13.8 mg/L	–	–
NO_2_	Low solubility (hydrolysis)	$2{\mathrm{NO}}_{2}+H_{2}O\to {\mathrm{HNO}}_{2}+{\mathrm{HNO}}_{3}$	0.90
NO_2_^−^	Hydrolysis/disproportionation at low pH	$3{\mathrm{HNO}}_{2}\to H_{3}O^{+}+{\mathrm{NO}}_{3}^{-}+2\mathrm{NO}$	–
H	–	–	2.30
F_2_	–	$1/2F_{2}+e\to F^{-}$	2.87
MnO_4_^−^	–	${\mathrm{MnO}}_{4}^{-}+8H^{+}+5e\to {\mathrm{Mn}}^{2+}+4H_{2}O$	1.52
Cl_2_	Insoluble	$1/2{\mathrm{Cl}}_{2}+e\to {\mathrm{Cl}}^{-}$	1.36
Fe_3_^+^	–	${\mathrm{Fe}}^{3+}+e\to {\mathrm{Fe}}^{2+}$	0.77

**Table 3:** Overview of studies dealing with plasma sterilization in liquid phase.

No	Microorganism	Inactivation Efficacy	Liquid	Plasma Source	Carrier Gas	Description	Reference
1	*E. coli*	~5 log reduction	Water	Pulsed corona discharge	–	Point-plane electrode system in water. V_p-p_: 25 kV, current_p-p_: 400 A, pulse duration: 1.8 µs, pulse frequency: 1–50 Hz, gap: 0.5–5 cm, energy per pulse: 2 J.	[195]
2	*E. coli*	~4 log reduction	Water	Pulsed DC discharge	–	Single- and double-layer multi-channel plasma discharge array in water. Voltage: 23 kV, pulse duration: 3 µs, current: 20 A.	[196]
3	*E. coli*	~10^5^ times reduction	Water	Pulsed discharge plasma	O_2_/N_2_ and O_2_/Ar	Plasma was generated using a planar high voltage electrode above the water surface. Gap: 12 mm, voltage: 27 kV, pulse frequency: 50 Hz, conductivity of water: 500 µS/cm, liquid volume: 900 mL, gas flow: 2.5 L/min.	[190]
4	*E. coli*	6–7 log reduction	Maximum recovery diluent (MRD), PBS	Atmospheric DBD plasma	Ambient air	Direct and indirect plasma treatment on the solutions. Voltage: 40 kV, treatment time: 10–300 s, gap: 10 mm (direct), 120–160 mm (indirect), liquid volume: 100 µL.	[176]
5	*E. coli*	~3 log reduction	Distilled water, saline	DC positive streamer discharge	–	Point-hollow plane electrode system was used to generate indirect plasma on the solutions. Liquid volume: 6.5 mL, treatment time: 1–9 min, gap: 6 mm, voltage: 15 kV.	[197]
6	*S. aureus*	2 log reduction	Distilled water	Atmospheric DC bubbling discharge	Ar	Plasma was generated in a quartz glass tube immersed in water. Gas flow: 2.5 L/h, gap: 20 mm, treatment time: 0–40 min, voltage: 6.5 kV, current: 150 mA, power: 0.4 W.	[188]
7	*E. coli*	7 log reduction	NAC	Atmospheric DBD plasma	Ambient air	Plasma was generated above the liquid. Liquid volume: 1 mL, treatment time: 1–3 min, gap: 2 mm, voltage: 31.4 kV, frequency: 15 kHz, power density: 0.29 W/cm^2^.	[194]
8	*E. coli*	7 log reduction	Distilled water	Atmospheric floating-electrode DBD plasma	–	Gap: 2 mm, liquid volume: 1 mL, treatment time: 0–3 min.	[189]
9	*E. coli*, *L. citreum*	More than 10^6^ times reduction	Distilled water	Pulsed plasma jet	He	Gap: 20 mm, liquid volume: 500 µL, gas flow: 2.0 L/min, frequency: 13.9 kHz, voltage: −3.5–5 kV, treatment time: 0–300 s.	[180]
10	*S. aureus*	100%	Water, water/LB culture media, water/bacteria suspension	Atmospheric DC micro-jet plasma	Air	Plasma was produced in the liquid. Gas flow: 2–3 slm, voltage: 400–600 V, current: 0–35 mA, liquid volume: 20 mL, treatment time: 0–20 min.	[186]
11	*P. aeruginosa*	–	Distilled water, saline	Atmospheric RF plasma jet	Ar	Plasma on the solutions. Gas flow: 1.5 slm, frequency: 13.56 MHz, power: 1.4 W, liquid volume: 100 µL, gap: 8 mm.	[191]
12	*S. aureus*, *E. faecalis*, *E. coli*, *P. aeruginosa*, MRSA, *M. terrae*, *M. abscessus*, *B. cereus*, *C. albicans*, *T. mentagrophytes*, *A. niger*	In case of *S. aureus* (for other cases please refer to the article): CO_2_ and N_2_: more than 6 log reduction at 60 s O_2_: 3 log reduction at 120 s Ar and mock air: less than 1 log reduction at 120 s	Bacterial suspension in PBS	AC Multi-gas plasma jet	CO_2_, N_2_, Ar, O_2_, mock air	Frequency: 16 kHz, voltage: 9 kV, power: 10 W, gas flow: 1 L/min, gap: 6 mm.	[192]
13	*E. coli*	100%	Deionized water	Pulsed DBD plasma	Air, O_2_	Liquid volume: 10 mL, gap: 1.5 mm, voltage: 17 kV, pulse frequency: 1.7 kHz, treatment time: 0–30 min.	[179]
14	*E. coli*	–	Tap water	Pulsed submerged arc discharge	–	Liquid volume: 50 mL, pulse frequency: 100 Hz, voltage: 80 V, pulse duration: 20 µs, treatment time: 5–60 s.	[198]
15	*E. coli*	Nearly no bacterial inactivation in Ar/ considerable reduction in air	Distilled water, NaCl	Surface DBD plasma	Ar, ambient air	Gap: 5 mm, gas flow: 0.5 slm, voltage: 10 kV (air), 3 kV (Ar), liquid volume: 5 mL, treatment time: 0–30 s.	[175]
16	*E. coli* and λDNA	100%	Citric acid buffer, TE buffer	Atmospheric pulsed plasma jet	He	Gas flow: 2 L/min, voltage: 15 kV, pulse frequency: 3 kHz, pulse duration: 2.8 µs, gap: 40 mm, liquid volume: 100 µL, treatment time: 0–120 s.	[199]
17	*E. coli*, *B. subtilis*, biofilms	100%	Physiological solution	DC pulsed streamer-spark discharge	–	Multi point-plane geometry was used to generate bubbling discharge. Gap: 10–15 mm, power: 30, 60 W, treatment time: 0–420 s.	[200]
18	*S. aureus*, Multidrug resistant bacteria	NPP: 4–5 log reduction DBD: 3 log reduction	Saline solution	Nano-second pulsed plasma and AC DBD surface plasma in Ar	–	NPP: Needle to needle electrode geometry in the liquid. Voltage: 6 kV, current: 0.7 kA. DBD surface plasma on the solution: Voltage: 0.6 kV, current: 14 mA, treatment time: 5 min.	[172]
19	*A. sanguinea*, *S. trochoidea*, *H. triquetra*	–	Algal culture	Remote DBD plasma	Air	Gap: 25 mm, voltage: 9 kV, frequency: 60 Hz, gas flow: 4 L/min, liquid volume: 1 mL, treatment time: 40–60 s.	[201]
20	*E. coli*, *B. atrophaeus*, *S. aureus*	–	Water, physiological saline, PBS	Pulsed surface DBD plasma	Ambient air	Indirect DBD plasma treatment. Gap: 5 mm, voltage: 10 kV, frequency: 20 kHz, liquid volume: 1.5, 5, 10 mL, treatment time: 0–30 min.	[173]
21	*E. coli*	5.5 log reduction	Physiological saline, PBS	Indirect AC DBD plasma	Air	Frequency: 10 kHz, voltage: 2–6 kV, power: 0.3–7 W, liquid volume: 150 µL, treatment time: 30–300 s.	[193]
22	*E. coli*	~6 log reduction	Water, buffered and non-buffered saline solutions	DC transient spark discharge	Air	Electro-spraying in point-plane electrode system. Gap: 10 mm, voltage: 14 kV, liquid flow rate: 0.5 mL/min, treatment time: 5 min.	[178]
23	*S. aureus*	More than 99% reduction	Water	Atmospheric DC plasma micro-jet	He/O_2_	Plasma immersed in water. Gas volume ratio: He/O_2_: 98/2%, gas flow: 2.5 slm, voltage: 400 V, current: 35 mA, treatment time: 0–16 min.	[187]
24	*E. coli*	~5 log reduction	Water	Pulsed discharge	Air, O_2_, N_2_	Multi-needle electrode system on the liquid. Liquid volume: 120 mL, gap: 4 mm, gas flow: 0.5–4 L/min, treatment time: 0–900 s, voltage: 17 kV.	[177]

**Table 4:** Different types of UV radiation and their energies.

Name	Wavelength (nm)	Energy (eV)
Ultraviolet A (UVA)	400–315	3.94–3.10
Ultraviolet B (UVB)	315–280	4.43–3.94
Ultraviolet C (UVC)	280–200	6.20–4.43
Vacuum ultraviolet (VUV)	200–100	12.4–6.20

**Table 5:** Hydrogen peroxide (H_2_O_2_) formation reactions [175,208,209].

Reactants		Products		
${\mathrm{OH}}^{•}+{\mathrm{OH}}^{•}$	→	$H_{2}O_{2}$		
$2H_{2}O$	→	$2H_{2}+O_{2}$	→	$H_{2}+H_{2}O_{2}$
$2{\mathrm{HO}}_{2}$	→	$H_{2}O_{2}+O_{2}$		
${\mathrm{HO}}_{2}+H_{2}O$	→	$H_{2}O_{2}+\mathrm{OH}$		
$H_{2}+{\mathrm{HO}}_{2}$	→	$H_{2}O_{2}+H$		
$2{\mathrm{HOO}}^{•}$	→	$H_{2}O_{2}+O_{2}$		

**Table 6:** Bond dissociation energies for biologically relevant molecules [208,210].

Reaction	Bond Dissociation Energy		Dissociation Wavelength (nm)
	kJ/mol	eV	
$HO_{2}\to H+O_{2}$	197	2.04	607.8
NH→N+H	356	3.69	336
$H_{2}O_{2}\to H+HO_{2}$	374.5	3.88	319.5
OH→O+H	428	4.44	279.2
$H_{2}\to 2H$	435.9	4.52	274.3
$O_{2}\to 2O$	498.3	5.16	240.3
$H_{2}O\to OH+H$	498.7	5.17	239.8
$CO_{2}\to CO+O$	532.2	5.52	224.6
NO→N+O	631.6	6.55	189.3
$N_{2}\to 2N$	945.4	9.80	126.5
CO→O+C	1076.5	11.16	111.1

**Table 7:** Overview of studies dealing with plasma–biological liquid interactions envisioning cancer therapy.

No	Biological Target	Treatment Type	Liquid	Plasma Source	Carrier Gas	Description	Reference
1	Endothelial cells, vascular smooth muscle cells	Direct	Culture medium	Atmospheric RF plasma needle	He	RF glow discharge. Frequency: 13.56 MHz, gas flow: 2 L/min, liquid volume: 10–60 µL, gap: 3 mm, voltage: 226–280 V, treatment time: 30–540 s.	[243]
2	Human Melanoma skin cancer cells	Direct	Culture medium	FE-DBD plasma	Air	Voltage: 10–30 kV, gap: 3 mm, power: 4 W, liquid volume: 200 µL, treatment time: 5–30 s.	[242]
3	Endothelial cells, vascular smooth muscle cells	Direct	Culture medium	Atmospheric RF plasma needle	He	Indirect RF glow discharge. Frequency: 13.56 MHz, gas flow: 2 L/min, gap: 1 mm, treatment time: 2–50 s.	[244]
4	Blood	Direct	Blood	AC plasma spray	Air	Frequency: 60 Hz, power: 170 W.	[218]
5	Human bladder cancer cells	Direct	Culture medium	Pulsed DC plasma jet	He/O_2_	Gas flow: He/O_2_: 100, 500/5, 10, 20 sccm, voltage: 1.2–1.8 kV, frequency: 20, 50 kHz, liquid volume: 0.2–1 mL, treatment time: 10, 20 s, gap: 6 mm.	[246]
6	Mouse melanoma cancer cells	Direct	Culture medium	Cold surface-type DBD discharge	Ambient air	Voltage: 4.2 kV, power: 4.26 W, treatment time: 10, 30, 50 s, gap: 5 mm.	[247]
7	Mammalian breast epithelial cells	Direct and indirect	Culture medium	AC pulsed DBD plasma	Air	Voltage: 20 kV, pulse duration: 1.65 µs, rise time: 5 V/ns, gap: 2 mm, liquid volume: 100 µL.	[251]
8	Murine melanoma and fibroblast tumor cells	Direct	Culture medium	Micro-plasma jet employing a hollow-core glass optical fiber	He	Gas flow: 10 sccm, frequency: 32 kHz, voltage: 17 kV, gap: 5 mm, treatment time: 10 s.	[256]
9	Human ovarian cancer cells	Direct	Culture medium	Non-equilibrium atmospheric plasma	Ar	Gas flow: 2 slm, liquid volume: 2 mL, gap: 15 mm, treatment time: 30–300 s, voltage: 10 kV, frequency: 60 Hz.	[257]
10	Mouse epithelial ovarian cancer cells	Indirect	Culture medium	Non-equilibrium atmospheric plasma	Ar	Gas flow: 2 slm, liquid volume: 100 µL, gap: 15 mm, treatment time: 30–300 s, voltage: 10 kV, frequency: 60 Hz.	[258]
11	CRFK feline kidney cells	Indirect and direct	Physiological liquid	RF atmospheric plasma jet	Ar	Gas flow: 1.5 slm, frequency: 13.7 MHz, liquid volume: 100 µL.	[259]
12	Mouse liver epithelial cells	Indirect	Various culture media	Atmospheric plasma jet	Ar	Gas flow: 1.9 slm, frequency: 1.1 MHz, voltage: 2–6 kV, liquid volume: 100 µL, gap: 1 mm, treatment time: 60, 120 s.	[260]
13	Human U87 cancer cells, human MDA-MB-231 cancer cells, MCF-7 cancer cells	Indirect	Cell culture media	DC cold plasma jet	He	Gap: 3 cm, liquid volume: 1 mL, time: 0.5–2 min, gas flow: 4.7 L/min, input voltage: 11.5 V, output voltage: 3.16 kV, power: 5 W.	[261]
14	Human epithelial PC cell lines LNCaP and PC-3	Indirect	Sespended cells in RPMI medium	DC cold plasma jet	Ar	Liquid volume: 500 µL, time: 10 s, gas flow: 3 L/min, voltage: 65 V, frequency: 1.1 MHz.	[262]
15	Breast cancer cells	Indirect	Deionized water	Cold plasma jet	Ar, He, N_2_	Voltage: 2–5 kV, frequency: 30 kHz, gas flow: 0.3 L/min.	[249]
16	Human cervical carcinoma cell line HeLa, sarcoma U2OS cells, breast cancer cells	Indirect	DMEM cell culture medium	Micro-plasma jet	Air	Gas flow: 10 L/min, time: 5 min.	[263]
17	Human gastric cancer cells	Indirect	Deionized water	Cold plasma jet	Ar	Gas flow: 0.4 L/min, time: 5–30 min, voltage: 8 kV, average current: 0.23 mA, frequency: 6.25 kHz.	[264]

**Table 8:** An overview of studies on the impact of non-thermal plasma on liquid food products.

No	Food Product	Investigated Microorganism	Plasma Source	Carrier Gas	Description	Reference
1	Commercial UHT and raw milk	*E. coli*	Atmospheric AC corona discharge	Air	Plasma inside the solution. Voltage: 9 kV, current: 90 mA, time: 3–30 min.	[294]
2	Milk	*E. coli*, L. monocytogenes, S. typhimurium	Atmospheric DBD plasma	Air	Power: 250 W, frequency: 15 kHz, time: 5, 10 min.	[295]
3	Hen egg	Hen egg white lysozyme in aqueous solution	Low frequency atmospheric AC plasma jet	He/O_2_	Plasma inside the solution. Gas flow: He/O_2_ = 0.50/0.15 L/min, time: 3 min.	[296]
4	Milk	α-casein, β-lactoglobulin, α-lactalbumin proteins in PBS	Atmospheric RF plasma	Ar	Gas flow: 30.7 L/min, frequency: 13.56 MHz.	[297]
5	Tomato	Tomato peroxides, as a model enzyme	Atmospheric DBD plasma	Air	Gap: 26 mm, voltage: 30, 40, 50 kV, time: 1–5 min.	[298]
6	Fresh orange juice	Vitamin C, S. aureus, *E. coli*, C. albicans	Atmospheric DBD plasma	Air	Frequency: 60 kHz, gap: 3 mm, voltage: 20 kV, Power density: 1.14 W/cm^2^, time: 3–25 s.	[299]
7	Peptone solution	*E. coli*, S. aureus, S. enteritidis, B. cereus	Atmospheric pulsed bubble discharge	N_2_, CO_2_, Air	Pulse rate: 320 pps, voltage: 28 kV, pulse width: 90 ns, time: 50 s, gas flow: 10 L/min.	[288]
8	Spinach	F. oxysporum	Atmospheric barrier-type surface discharge plasma	Air, O_2_, N_2_, He, Ar	Pulsed plasma directly into the solution. Voltage: 16 kV, pulse width: 20 µs, pulse cycle: 200 µs, gas flow: 500 mL/min, time: 5–50 min.	[293]
9	Emulsion-type sausage	Plasma-treated water as a nitrate source	Atmospheric surface DBD plasma	Air	Frequency: 15 kHz.	[300]
10	Raw milk	Lipid composition of milk	Atmospheric AC corona discharge	Air	Time: 3–20 min, gap: 8 mm, current: 90 mA, voltage: 9 kV.	[301]
11	Pomegranate juice	Anthocyanins and color	Atmospheric plasma jet	Ar	Time: 3, 5, 7 min, juice volume: 3, 4, 5 cm^3^, gas flow: 0.75, 1, 1.25 L/min, gap: 1.5 cm, frequency: 25 kHz, power: 6 W, voltage: 2.5 kV, current: 3 mA.	[302]
12	Tomato juice	Volatile components	Atmospheric bubbling discharge	Air	Voltage: 10 kV, time: 5 min.	[303]
13	Chokeberry juice	Stability of hydroxycinnamic acids, flavonols and anthocyanins	Atmospheric plasma jet	Ar	Frequency: 25 kHz, power: 4 W, gas flow: 0.75 L/min, juice volume: 3, 5, 7 cm^3^, time: 3, 5 min, gap: 1.5 cm.	[304]
14	Commercial pasteurized and raw milk	*E. coli*	Atmospheric nanosecond pulsed bubble discharge	Ar	A gas bubble discharge was generated inside milk using a needle to plate electrode configuration, immersed in milk. Frequency: 2.5, 4 kHz, time: 75–120 s, gap: 1 cm, pulse width: 10 ns, voltage: 9 kV.	[305]
15	Sour cherry juice	Content of anthocyanins and phenolic acids	Atmospheric plasma jet	Ar	Frequency: 25 kHz, power: 4 W, gas flow: 0.75, 1, 1.25 L/min, juice volume: 2, 3, 4 mL, time: 3, 4, 5 min, gap: 1.5 cm, voltage: 2.5 kV, current: 3 mA.	[306]

**Table 9:** Overview of studies dealing with plasma electrical discharges in dielectric media.

No	Type of Oil	Electrode Geometry	Streamer Polarity	Gap (cm)	Description	Reference
1	Commercial mineral transformer oil	Point-plane	Negative	0.5–35	Grounded plane in the oil. Voltage: 460 kV.	[328]
2	Mineral oil	Point-plane	Positive	2.5–30	Under impulse voltage. System bandwidth ~35 MHz, voltage: 500 kV, impulses: 0.4/1400 µs.	[321]
3	Transformer oil	Point-plane	Negative, positive	0.3	Pulse duration: 0–10 µs, voltage: 50, 70 kV.	[319]
4	Pharmaceutical grade white oil	Point-plane	Negative, positive	8	Bush-like filamentary streamers. Voltage: 0–500 kV.	[334]
5	Mineral oil	Point-plane	Positive	5–10	High voltage Impulses: 0.4/1400 µs, voltage: 102, 138, 184 kV.	[341]
6	Cyclohexane with pyrene additive	Point-plane	Positive	0.6, 5	Voltage: 4–450 kV, high voltage Impulses: 0.4/1400 µs.	[331]
7	Silicone oil, transformer oil	Point-plane	Positive	0.2–0.3	Voltage: 24 kV.	[314]
8	Mineral oil	Point-plane	Positive	2–10	Voltage: 104–352 kV.	[326]
9	Insulating oil	Point-point, point-plane	Negative, positive	Point-point: 15–30, 45–60 Point-plane: 15–45, 60–100	Frequency of applied voltage: 50 Hz, lightning impulse voltage duration: 1/40 µs, switching impulse voltage duration: 600/3600 µs.	[342]
10	Transformer oi	Sphere-plane	Negative, positive	30, 60	AC voltage: 800 kV, duration of the discharge: 50–100 ns.	[343]
11	Insulating oil	Point-plane	Negative, positive	1–7	Strong non-uniform field at voltages up to 700 kV.	[323]
12	Transformer oil	Point-plane	Positive	5–20	Under step and AC voltages. Positive impulse: 1.2/700 µs, voltage: 500 kV.	[324]
13	Oil	Point-plane	Negative, positive	7.62	Impulsive 1.5/40 µs wave. Crest voltage for positive streamer: 190 kV, for negative streamer: 240 kV.	[318]
14	Silicon oil	–	–	–	60 MHz (HF)/2 MHz (LF) dual frequency capacitively coupled plasma. Power: 165 W and 100 W, base pressure: 0.5 mPa, work pressure: 50 Pa, discharge gas: C_2_F_6_ and CHF_3_, gas flow: 30 sccm, time: 30 min.	[344]
14	Mineral oil	Point-plane	Negative, positive	10, 20, 35	Under impulse voltage. Positive impulse: 1.2/1400 µs, voltage: 470 kV	[327]
15	Synthetic and natural ester transformer liquids	Point-plane	Negative, positive	1.5–10	Standard lightening impulse duration: 1.2/50 µs, voltage: 60, 80, 110, 230, −70, −110, −210, −290, −320 kV.	[336]
16	Mineral oil	point-plane, sphere-plane	Negative, positive	10, 30, 60, 80	AC voltage: 325, 625 kV.	[325]
17	Natural ester liquid	Point-plane	Negative, positive	2–20	Voltage up to 460 kV, impulse: 0.5/1400 µs.	[335]
18	Commercial naphthenic transformer oil	Point-plane	Negative, positive	6.7	Voltage for positive point: 188, 165 kV, for negative point: 300 kV, impulse: 1/180 µs.	[320]
19	Brazilian heavy crude oil containing emulsified water	–	–	2	DBD plasma using different gases: CO_2_, H_2_ and natural gas. Time: 1, 1.5, 4 h, voltage: 13 kV.	[340]
20	Heavy oil and cokes from petrochemical refinery processes	–	–	5	2.45 GHz microwave cold water plasma. Time: 30 min, power: 80 W.	[337]
21	Heavy oil	Packed-bed, plate-plate	–	Packed-bed: 5–25 Plate-plate: 0.3–1	Packed-bed reactor: 60 Hz AC voltage for Ar spark discharge, gas flow: 50–400 mL/min, power: 5–25 W. Plate-plate reactor: 50/60 Hz AC current, voltage: 9 kV, Ar gas flow: 50–400 mL/min, power: 3–12 W.	[338]
22	Heavy oil	Plate-plate	–	0.3–1	60 Hz AC voltage 9 kV, Ar flow rate: 225 mL/min, power: 3–12 W.	[339]

## Abbreviations

AC	Alternating current
CHL	Chloroform
DBD	Dielectric barrier discharge
DC	Direct current
DCM	Dichloromethane
DMA	*N*,*N*-Dimethylaniline
DMF	*N*,*N*-Dimethylformamide
DPBS	Dulbecco’s phosphate buffered saline
EEM	Excitation-emission matrix
ELCAD	Electrolyte-cathode discharge
EPR	Electron paramagnetic resonance
FE-DBD	Floating electrode dielectric barrier discharge
FGF	Fibroblast growth factor
GC-MS	Gas chromatography-mass spectroscopy
GDE	Glow discharge electrolysis
HBSS	Hank’s balanced salt solution
ICCD	Intensified charge-coupled device
ICP	Inductively coupled plasma
IMDM	Iscove’s modified Dulbecco’s medium
LIBS	Laser-induced breakdown spectroscopy
LTE	Local thermodynamic equilibrium
MALDI	Matrix-assisted laser desorption ionization
MW	Microwave
NAC	N-acetylcysteine
NMR	Nuclear magnetic resonance
NP	Nanoparticle
NPP	Nano-pulsed plasma
OES	Optical emission spectroscopy
PAN	Polyacrylonitrile
PAW	Plasma-activated water
PBS	Phosphate buffered saline
PCL	Poly-ε-caprolactone
PEO	Polyethylene oxide
PEPT	Pre-electrospinning plasma treatment
PLA	Poly lactic acid
PLIs	Plasma-liquid interactions
PLLA	Poly-L-lactic acid
PVP	Polyvinyl pyrrolidone
RF	Radiofrequency
RNS	Reactive nitrogen species
RONS	Reactive oxygen and nitrogen species
ROS	Reactive oxygen species
RPMI	Roswell Park Memorial Institute
SCCM	Standard cubic centimeter per minute
SEM	Scanning electron microscopy
SFM	Serum-free medium
SPB	Sørensen’s phosphate buffer
TCE	Trichloroethylene
UHT	Ultrahigh temperature
UV	Ultraviolet
XPS	X-ray photoelectron spectroscopy
